# Complete Genome Sequencing and Targeted Mutagenesis Reveal Virulence Contributions of Tal2 and Tal4b of *Xanthomonas translucens* pv. undulosa ICMP11055 in Bacterial Leaf Streak of Wheat

**DOI:** 10.3389/fmicb.2017.01488

**Published:** 2017-08-10

**Authors:** Nargues Falahi Charkhabi, Nicholas J. Booher, Zhao Peng, Li Wang, Heshmat Rahimian, Masoud Shams-Bakhsh, Zhaohui Liu, Sanzhen Liu, Frank F. White, Adam J. Bogdanove

**Affiliations:** ^1^Plant Pathology and Plant-Microbe Biology Section, School of Integrative Plant Science, Cornell University, Ithaca NY, United States; ^2^Department of Plant Pathology, Tarbiat Modares University Tehran, Iran; ^3^Department of Plant Pathology, Kansas State University, Manhattan KS, United States; ^4^Department of Plant Pathology, University of Florida, Gainesville FL, United States; ^5^Department of Plant Protection, Sari Agricultural Science and Natural Resources University Sari, Iran; ^6^Department of Plant Pathology, North Dakota State University, Fargo ND, United States

**Keywords:** TAL effectors, SMRT sequencing, comparative genomics, mutational analysis, bacterial diseases of crop plants

## Abstract

Bacterial leaf streak caused by *Xanthomonas translucens* pv. undulosa (Xtu) is an important disease of wheat *(Triticum aestivum) and barley (Hordeum vulgare)* worldwide. Transcription activator-like effectors (TALEs) play determinative roles in many of the plant diseases caused by the different species and pathovars of *Xanthomonas*, but their role in this disease has not been characterized. ICMP11055 is a highly virulent Xtu strain from Iran. The aim of this study was to better understand genetic diversity of Xtu and to assess the role of TALEs in bacterial leaf streak of wheat by comparing the genome of this strain to the recently completely sequenced genome of a U.S. Xtu strain, and to several other draft *X. translucens* genomes, and by carrying out mutational analyses of the TALE *(tal)* genes the Iranian strain might harbor. The ICMP11055 genome, including its repeat-rich *tal* genes, was completely sequenced using single molecule, real-time technology (Pacific Biosciences). It consists of a single circular chromosome of 4,561,583 bp, containing 3,953 genes. Whole genome alignment with the genome of the United States Xtu strain XT4699 showed two major re-arrangements, nine genomic regions unique to ICMP11055, and one region unique to XT4699. ICMP110055 harbors 26 non-TALE type III effector genes and seven *tal* genes, compared to 25 and eight for XT4699. The *tal* genes occur singly or in pairs across five scattered loci. Four are identical to *tal* genes in XT4699. In addition to common repeat-variable diresidues (RVDs), the *tal* genes of ICMP11055, like those of XT4699, encode several RVDs rarely observed in *Xanthomonas*, including KG, NF, Y^∗^, YD, and YK. Insertion and deletion mutagenesis of ICMP11055 *tal* genes followed by genetic complementation analysis in wheat cv. Chinese Spring revealed that Tal2 and Tal4b of ICMP11055 each contribute individually to the extent of disease caused by this strain. A largely conserved ortholog of *tal2* is present in XT4699, but for *tal4b*, only a gene with partial, fragmented RVD sequence similarity can be found. Our results lay the foundation for identification of important host genes activated by Xtu TALEs as targets for the development of disease resistant varieties.

## Introduction

*Xanthomonas* is a genus of plant associated and plant pathogenic, Gram-negative bacteria. *Xanthomonas* species infect approximately 124 monocot species and 268 dicot species and cause economic losses in many crops and ornamentals (Leyns et al., 1984; Chan and Goodwin, 1999; Büttner and Bonas, 2002; Parkinson et al., 2009). Among the molecular determinants that contribute to the complex process of pathogenesis and to host adaptation, type III secreted effector proteins play a key role (Büttner and Bonas, 2010; Jacques et al., 2016). Many such proteins have been identified in plant pathogenic species and pathovars (pathogenic variants of a species that have distinctive host or tissue specificity) of *Xanthomonas*. Among the best-characterized are the transcription-activator like effector (TALE) proteins, which localize to the nucleus following injection from the bacteria into plant cells (for reviews, see Bogdanove et al., 2010; Schornack et al., 2013). TALEs have been identified in many though not all species and pathovars of *Xanthomonas*, and closely related proteins, called RipTALs, have been identified in strains of *Ralstonia solanacearum* (Schornack et al., 2006; Heuer et al., 2007; White et al., 2009). In the plant cell nucleus, TALEs individually activate specific corresponding host genes by binding to effector-specific effector binding elements (EBEs) in the promoters. Their DNA binding specificity is conferred by a central repeat region (CRR), which is flanked by an N-terminal domain that contains a type III secretion signal, and a C-terminal domain that harbors nuclear-localization signals and an acidic activation domain. The CRR contains tandem repeats of 33–35 amino acids polymorphic at residues 12 and 13, referred to as the repeat-variable diresidue (RVD). Each repeat interacts with a single base in the EBE, contiguously, determined by the RVD through base-specific contacts with the residue 13 side chain (Boch and Bonas, 2010; Bogdanove et al., 2010). RipTALs display some differences from TALEs; they harbor RVDs not found or found only rarely so far in TALEs, RipTAL repeats are 35 aa in length and exhibit greater overall polymorphism outside the RVD than TALEs, and RipTALs bind best to EBEs immediately preceded (5′) by a guanine rather than the thymine commonly found upstream of a TALE EBE (Doyle et al., 2013; Lange et al., 2013).

Based on a handful of characterized examples, TALEs, in general, are thought to contribute to symptom development and bacterial growth by inducing host susceptibility (*S*) genes. In *Xanthomonas oryzae* pv. oryzae, the agent of bacterial leaf blight of rice, TALEs PthXo1 and PthXo2 induce the rice susceptibility genes *OsSWEET11* and *OsSWEET13*, respectively (Yang et al., 2006; Zhou et al., 2015), and AvrXa7, PthXo3, TalC, and Tal5 promote the expression of *OsSWEET14* (Yang and White, 2004; Antony et al., 2010; Römer et al., 2010; Yu et al., 2011; Streubel et al., 2013). In the absence of *SWEET* gene expression, strains of *X. oryzae* pv. oryzae show reduced ability to colonize rice. Other TALEs linked to virulence include PthXo6 and PthXo7, which induce the expression of transcription factor genes *OsTFX1* and *OsTFIIA*γ*1*, respectively (Sugio et al., 2007). Tal2g of *X. oryzae* pv. oryzicola strain BLS256 contributes to the expansion of water soaked lesions and to the egress of bacteria to the leaf surface in bacterial leaf streak of rice by activating a putative sulfate transporter gene (Cernadas et al., 2014). *X. citri* ssp. *citri* and *X. fuscans* ssp. *aurantifolii* strains, which cause citrus canker, have one unique TALE each that activates *CsLOB1*, a member of the lateral organ boundaries family of regulatory genes that control complex phenotypes, including cell hypertrophy, hyperplasia and rupture of the epidermis (Hu et al., 2014). TAL20 found in strains of *X. axonopodis* pv. manihotis strains, the agent of cassava bacterial leaf blight, induces the sugar transporter *MeSWEET10a* to promote disease development (Cohn et al., 2014). Similarly, Avrb6 of *X. citri* pv. manihotis contributes to symptom development in bacterial blight of cotton by activating the *GhSWEET10* gene (Cox et al., 2017). AvrHah1 of *X. gardneri* was shown to activate expression of a bHLH transcription factor that positively regulates a pectate lyase gene involved in water soaking in bacterial spot of tomato (Schwartz et al., 2017).

Some TALEs induce host resistance by activating genes that cause rapid host cell death and restrict pathogen growth. AvrBs3, from *X. euvesicatoria*, and AvrXa27, AvrXa10, and AvrXa23, all of *X. oryzae* pv. oryzae, activate the pepper *Bs3* gene for resistance to bacterial spot and the *Xa27, Xa10*, and *Xa23* genes for resistance to bacterial blight of rice, respectively (Gu et al., 2005; Römer et al., 2007; Tian et al., 2014; Wang et al., 2015). Some TALEs trigger disease resistance independent of direct host gene activation, likely through protein–protein interaction (Schornack et al., 2004; Read et al., 2016; Triplett et al., 2016), and recently, a number of truncated TALEs of *X. oryzae* pv. oryzae and *X. oryzae* pv. oryzicola have been shown to function as suppressors of that type of resistance (Ji et al., 2016; Read et al., 2016).

Despite the demonstrated importance of several TALEs in different *Xanthomonas* species, *tal* genes are unevenly distributed within the genus, and even within a species or pathovar the numbers of *tal* genes can vary from more than two dozen to zero (Boch and Bonas, 2010). In species such as *X. oryzae* that have large numbers of *tal* genes, many within a strain appear not to play an important functional role (Yang and White, 2004). It has been speculated that such *tal* genes confer advantage by allowing, through recombination, rapid adaptation to host genotypic variation (Bogdanove et al., 2010).

Compared in a multi-locus sequence analysis using four housekeeping genes, *Xanthomonas* species cluster into two groups (Young et al., 2008). Group I, the smaller group, contains *X. albilineans, X. sacchari, X. theicola, X. hyacinthi*, and *X. translucens*. Group II contains the majority of described species, including *X. oryzae, X. citri, X. campestris, X. axonopodis*. Pathovars of the species *X. translucens*, in group I, are collectively pathogenic to diverse host plant species, including grain crop species (Bragard et al., 1997). Pathovars that infect grain crop species cause particularly important losses. These include: *X. translucens* pv. cerealis (Xtc), pathogenic to wheat, oat, rye, and bromus; pv. translucens (Xtt), pathogenic to barley; and pv. undulosa (Xtu), which displays broad host specificity, infecting wheat, barley oat, rye, bromus, and triticale (Bragard et al., 1997; Duveiller et al., 1997). Bacterial leaf streak (BLS) of wheat (*Triticum aestivum* L.) caused by Xtu and Xtc results in significant losses in many parts of the world (Boosalis, 1952; Schaad and Forster, 1985; El Attari et al., 1996; Milus et al., 1996; Adhikari et al., 2012). Lesions, which are typically water soaked and translucent initially, expand and coalesce into larger necrotic streaks (Mehta, 1990). Under humid conditions they may exude bacteria, which form droplets on the leaf surface (Mehta, 1993). Yield losses as high as 40% have been documented (Schaad and Forster, 1985), and the disease can also reduce grain quality (Mehta, 1990).

Draft genome sequence assemblies for several *X. translucens* strains, including strains of Xtc, Xtt, Xtu, and others, have been published (Wichmann et al., 2013; Pesce et al., 2015; Peng et al., 2016; Langlois et al., 2017), but, assembled from short reads, the assemblies do not include the repeat-rich, TALE-encoding sequences (*tal* genes). Recently, a complete genome assembly from the U.S. Xtu strain XT4699 was generated using single molecule, real-time (SMRT) technology (Pacific Biosciences), revealing eight TALE genes, and sequences of several *tal* genes individually cloned from other Xtu strains were also obtained, but none was functionally characterized (Peng et al., 2016). Likewise, two TALE sequences were found in the draft genome of Xtc strain CFBP 2541 and confirmed by amplicon sequencing (Pesce et al., 2015), and a whole genome sequence of Xtt strain DSM 18974 yielded eight TALE sequences (Jaenicke et al., 2016), but none of these were functionally characterized either.

*Xanthomonas translucens* pv. undulosa ICMP11055 is a highly virulent strain isolated from wheat cv. Tabasi in Kerman, Iran, in 1983 (by H.R.). Tested on wheat, barley, rye, and bromus, the strain was found pathogenic to each, causing characteristic, prominent water-soaking streaks with honey-like exudates (Alizadeh and Rahimian, 1989; N.F., unpublished). To better understand genetic diversity of Xtu and to assess the role of TALEs in bacterial leaf streak of wheat, we completely sequenced the ICMP11055 genome and compared it to that of the United States strain XT4699, which is known to infect barley, wheat, and triticale, and is moderately virulent in comparison to other United States strains (Z.L., unpublished), and to several other draft or complete *X. translucens* genomes. We then carried out mutational analysis of the *tal* genes found in ICMP11055 to test their contributions to virulence.

## Materials and Methods

### Bacterial Strains, Plasmids, and Primers

Bacterial strains and plasmids used in this study are listed in Supplementary Table S1, and primers used are provided in Supplementary Table S2. *Escherichia coli* strains were grown in LB medium at 37°C, and *X. translucens* strains were cultured in GYE (20 g/l glucose, 10 g/l yeast extract) at 28°C. Plasmids were introduced into *E. coli* and *X. translucens* by heat shock and electroporation, respectively. Antibiotics used for selection were as follows: ampicillin at 100 μg/ml, spectinomycin at 25 μg/ml, kanamycin at 25 μg/ml for *E. coli* and 50 μg/ml for *X. translucens* and tetracycline at 10 μg/ml for *E. coli* and 2 μg/ml for *X. translucens*.

### DNA Extraction

For SMRT sequencing, total genomic DNA of strain ICMP11055 was extracted from 30 ml of bacterial culture grown in GYE for 48 h at 28°C on a rotary shaker at 250 rpm as follows (modified from Ausubel et al., 1994). Bacterial suspension was harvested at 3,220 × *g* for 10 min at 4°C. The pellet was washed twice in 20 ml NE buffer (0.15 M NaCl/ 50 mM EDTA) and resuspended in 2.5 ml of 50 mM Tris pH 8.0, 50 mM EDTA. The suspension was frozen at -20°C and thawed at room temperature. Then, a solution of 0.5 ml 25 mM Tris pH 8.0, 10 μl ReadyLyse, and 50 μl RNase A (10 mg/ml) was added to the suspension and mixed thoroughly. Following incubation for 45 min on ice, one ml of STEP (0.5% SDS, 50 mM Tris pH 7.5, 40 mM EDTA, 2 mg/ml protease K) was added to the sample, and the sample was shaken well and incubated at 37°C for 1 h, mixing every 10–15 min. Then, 1.8 ml 7.5 M ammonium acetate was added and mixed gently. An equal volume phenol/chloroform (1:1 V/V) was added to each tube and mixed well by repeated inversions for 20 min. Following centrifugation at 7,200 × *g* for 10 min, the aqueous phase was transferred to a new tube and chloroform/isoamyl alcohol extraction was repeated twice more. The final aqueous phase was transferred to a sterile tube, and DNA was precipitated by adding two volumes of cold 95% ethanol and centrifugation at 1,800 × *g* for 5 min. The pellet was washed with 70% ethanol, dried at room temperature, and then resuspended in TE (10 mM Tris/HCl, 1 mM EDTA, pH 8.0).

To check for the existence of small plasmids, DNA was extracted and examined by agarose gel electrophoresis as described (Chakrabarty et al., 2010). *X. axonopodis* pv. vesicatoria strain 85-10 (Thieme et al., 2005) and *X. oryzae* pv. oryzicola BLS256 (Bogdanove et al., 2011) were used as positive and negative controls, respectively.

For PCR analysis of Xtu ICMP11055 mutant strains, genomic DNA was extracted using the GenElute Bacterial Genomic Kit (Sigma–Aldrich, St. Louis, MO, United States). PCR was conducted in 20-μl volume using Phire Hot Start II DNA Polymerase (Thermo Fisher Scientific, Waltham, MA, United States).

Routine plasmid isolation from *E. coli* was carried out using the E.Z.N.A.^®^ Plasmid DNA Mini Kit (Omega Bio-Tek, Norcross, GA, United States).

### Genome Sequencing, Assembly and Annotation

The ICMP11055 genome was sequenced to 120× coverage using two SMRT cells and the P5-C3 chemistry. *De novo* assembly was done using the HGAP (Chin et al., 2013) v3.0 software package. The assembly was verified with an independent assembly of the TALE gene regions with the PBX toolkit as described (Booher et al., 2015) and annotated by NCBI with the NCBI Prokaryotic Genome Annotation Pipeline (PGAP). The *tal* genes were named according to the scheme described by Salzberg et al. (2008). The ICMP11055 genome sequence is available in GenBank under accession PRJNA264445.

### Phylogenetic Tree Construction Using Whole Genome SNP Analysis

Whole-genome discovery of single nucleotide polymorphisms (SNPs) and phylogenetic tree construction was carried out using the XT4699 genome sequence as the reference as described by Peng et al. (2016).

### Identification of Type III Effector Genes

Type III effector genes were identified as described by Peng et al. (2016), using blastp against the protein sequences derived from the NCBI annotation of the ICMP11055 genome and tblastn against the whole genome sequence.

### Cloning of ICMP11055 *tal* Genes

Clones of *tal1, tal4a*, and *tal4b* were obtained using a shotgun cloning approach, as described by Cernadas et al. (2014) with minor modifications. *Sph*I sites flank the CRR of most of the ICMP110055 *tal* genes, at short distances from the start and end of the CRR. Ten micrograms of genomic DNA of ICMP11055 were digested with *Sph*I. DNA fragments from 1.5 to 3 kb were then gel purified using 1% agarose and ligated into *Sph*I-linearized and alkaline phosphatase (Calf Intestinal; New England Biolabs, Ipswitch, MA, United States)-treated pTAL1 (Cermak et al., 2011). pTAL1 carries the *tal1c* gene of *X. oryzae* pv. oryzicola strain BLS256 missing the *Sph*I fragment that encompasses its CRR. Ligation products were introduced into *E. coli* TOP10 cells by electroporation, and clones carrying the central *Sph*I fragment of an ICMP110055 *tal* gene in the correct orientation were identified by colony PCR using oligonucleotide primers 1571 and 1593, which target sequences immediately 5′ of the CRR and within the *Sph*I fragment that are conserved across the ICMP11055 *tal* genes. The PCR conditions were as follows: initial denaturation at 98°C for 30 s, followed by 30 cycles of 98°C for 5 s, 62°C annealing for 5 s, 72°C extension for 1 min/kb and a final extension of 1 min at 72°C. The cloned *tal* gene fragments were identified based on their size and 5′ and 3′ sequencing with primers B235 and B236, respectively. Because the *Sph*I site downstream of the CRR is at a different location in the ICMP110055 *tal* genes from that in *tal1c* of BLS256, the ligation resulted in loss of a small stretch of coding sequence. This was corrected in each clone by replacing an *Aat*II to *Bpu*10I fragment spanning the ligation junction with the corresponding fragment from pTAL1. Each clone was confirmed by Sanger sequencing.

Clones equivalent to *tal2, tal3a, tal3b*, and *tal5* were assembled into pTAL1 using the Golden Gate kit of Cermak et al. (2011). RVDs NN and NG, already represented among the repeat modules of the kit, were used in place of HN and KG, because they show the same respective nucleotide specificities (Yang et al., 2014). For RVDs Y^∗^ and YK, new repeat modules were created using the Q5^®^ Site-Directed Mutagenesis Kit (New England Biolabs) and NN repeat modules as templates. Similarly, repeat module QD5 was created using HD5 as template. A last (truncated) repeat module for RVD QD (LR-QD) was synthesized and cloned in plasmid pCR8 as described (Cermak et al., 2011).

For expression in *Xanthomonas*, all reconstituted *tal* genes in pTAL1 were introduced into pKEB31 (Cermak et al., 2011) using Gateway LR Clonase (Life Technologies, Carlsbad, CA, United States) (see Supplementary Table S1).

### Mutagenesis of ICMP11055 *tal* Genes

The suicide plasmid pSM7 (Makino, 2005) was used to make a library of *tal* gene knockout strains of ICMP11055 by targeted integration. pSM7 carries a 4.5-kb *Pst*I fragment with all but the first 80 bp of *tal* gene aB4.5 (Bai et al., 2000) disrupted in the CRR by an insertion of the EZ-Tn5, NotI/KAN-3 transposon (Epicenter). Insertion endpoints in kanamycin resistant transformants were investigated by sequencing the distal ends of PCR amplicons extending in either direction from the transposon to the regions flanking the CRR. The primers used for amplifying the 5′ flanking DNA were primer 1527 (forward), matching a conserved 5′ sequence in Xtu *tal* genes, and primer 395 (reverse), corresponding to the 3′ end of the transposon. For the 3′ fragment, forward primer 397, corresponding to the 5′ end of the transposon, and reverse primer 398, originally designed based on a conserved 3′ sequence of *X. oryzae tal* genes, were used. The amplicon ends farthest from the CRR were sequenced using *tal* gene primers 1527 and 398, respectively. This process yielded a knockout mutant for *tal1, tal2*, and *tal3b*. For each, the 5′ and 3′ PCR product sequences indicated integration by a single crossover at the 5′ end of the *tal* gene.

For *tal3a* and for *tal4a* and *tal4b*, plasmid pK18mob::sacB (Schäfer et al., 1994) was used to generate a marker-free deletion mutant of the tal3 cluster (containing *tal3a* and *tal3b*) and separately a mutant with the tal4 cluster (containing *tal4a* and *tal4b*) deleted, following the approach of Kvitko and Collmer (2011). For the tal3 cluster, a unique 1000-bp upstream flanking sequence and a unique 624-bp downstream flanking sequence were amplified by PCR using the primer sets 1623/1624 and 1627/1628, respectively (Supplementary Figure S1 and Table S2). Following verification of the upstream and downstream fragments through sequencing by the use of the primers 1625 and 1629 (Supplementary Figure S1 and Table S2), the fragments were digested with *Sma*I and then ligated together using T4-DNA ligase. The ligation product was separated on 1% agarose by electrophoresis, and the gel-purified fragment was digested with *Eco*RI-HF and *Xba*I and cloned into the suicide plasmid pK18mob::sacB digested with the same enzymes. The recombinant plasmid was electrotransferred into ICMP11055. Transformants were plated on GYE containing kanamycin, and two colonies were cultured overnight without selection and then plated on GYE agar containing 10% sucrose. Resulting colonies were screened by colony-PCR using the 1626/1630 primer pair (Supplementary Figures S1, S2A). PCR products were verified via sequencing using primers 1625 and 1629 and the corresponding mutant tested to confirm kanamycin sensitivity. One of the confirmed mutants was chosen for further study and designated as Δtal3. A tal4 cluster deletion mutant, designated as Δtal4, was obtained in the same way, using primer sets 1631/1632 and 1635/1636 to amplify flanking sequence, primers 1633 and 1637 to sequence-confirm those fragments, primer set 1634/1638 to screen sucrose-resistant colonies, and primers 1633 and 1637 to sequence-confirm the PCR products resulting from the screening (Supplementary Figures S1, S2B).

A *tal5* deletion mutant was generated by marker exchange as follows. The chloramphenicol resistance gene was amplified from p*dcas9* (Bikard et al., 2013) using primer pair 1916/1917, *tal5* flanking sequences were amplified using primer sets 1914/1915 and 1918/1919, and the marker was cloned between the flanking sequences using NEBuilder^®^ HiFi DNA Assembly Cloning Kit (New England Biolabs) (Supplementary Figure S1). The construct was cloned into pK18mob::sacB using the NEBuilder High-Fidelity DNA Assembly Cloning kit (New England Biolabs). Following electrotransfer into ICMP11055, chloramphenicol- and sucrose-resistant but kanamycin-sensitive colonies were identified and the targeted mutagenesis confirmed by PCR using the primers 1877/1879 (Supplementary Figure S2C). One of the confirmed mutants was chosen for further study and designated as Δ*tal5*.

### Virulence Assays

Wheat cv. Chinese Spring was grown in a growth chamber under a cycle of 16 h light at 28°C and 8 h dark at 24°C with 75% relative humidity (seed kindly provided by M. Sorrells, Cornell University). Inoculum was prepared by suspending log-phase bacterial cells in sterile 10 mM MgCl_2_ to an optical density of 0.2 at 600 nm. Suspensions of wild-type cells and mutant cells (or, for complementation assays, mutants transformed with plasmid) were inoculated side by side across the midrib into the youngest two to three leaves of 3-week old plants by infiltration using a disposable syringe with a 30- or 31-gauge hypodermic needle inserted at a sharp angle into the leaf mesophyll tissue, from the adaxial side, and the edges of the infiltrated areas were marked using a permanent marker. For each mutant or transformed mutant, the expansion of lesions beyond the inoculated area was measured 9 days post-inoculation and results were expressed as the ratio of that length to the length resulting from the wild type inoculated opposite on the same leaf. Significant differences were determined using the paired Student’s *t-*test. The experiment was repeated twice.

## Results

### The *X. translucens* pv. undulosa ICMP11055 Genome

**FIGURE 1:**
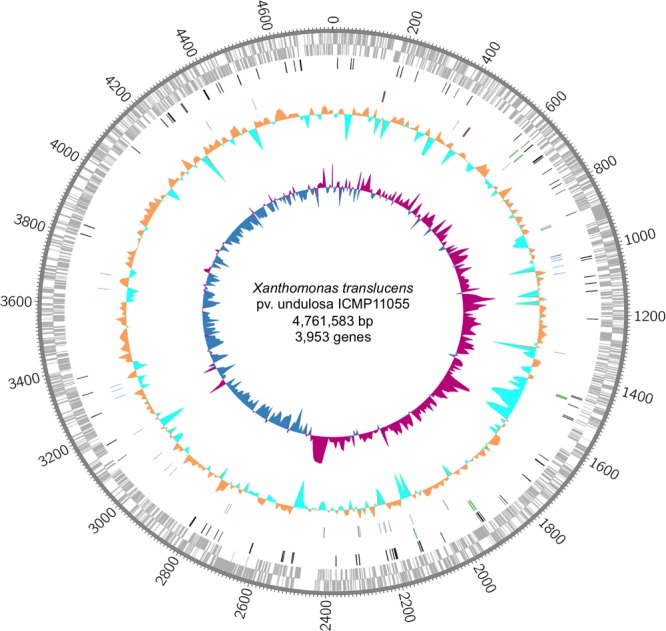
The *Xanthomonas translucens* pv. undulosa ICMP11055 genome. Rings, outermost to innermost, illustrate coordinates (kb), protein-coding genes on the forward (outer) and reverse strands, IS elements (black), *tal* genes (green), tRNA (blue) and rRNA (red) genes, percent GC content, and GC skew. GC skew illustrates (G–C)/(G+C) in 10 kb windows; positive values indicate the leading strand of replication, and negative values indicate the lagging strand.

The ICMP11055 genome was sequenced using SMRT sequencing. It consists of a single circular chromosome of 4,561,583 bp (**Figure 1** and Supplementary Figure S3). The genome has 67.8% average GC content and contains 3,953 annotated protein-encoding genes, 54 tRNA genes, and two rRNA operons, similar to other *Xanthomonas* genomes.

**FIGURE 2:**
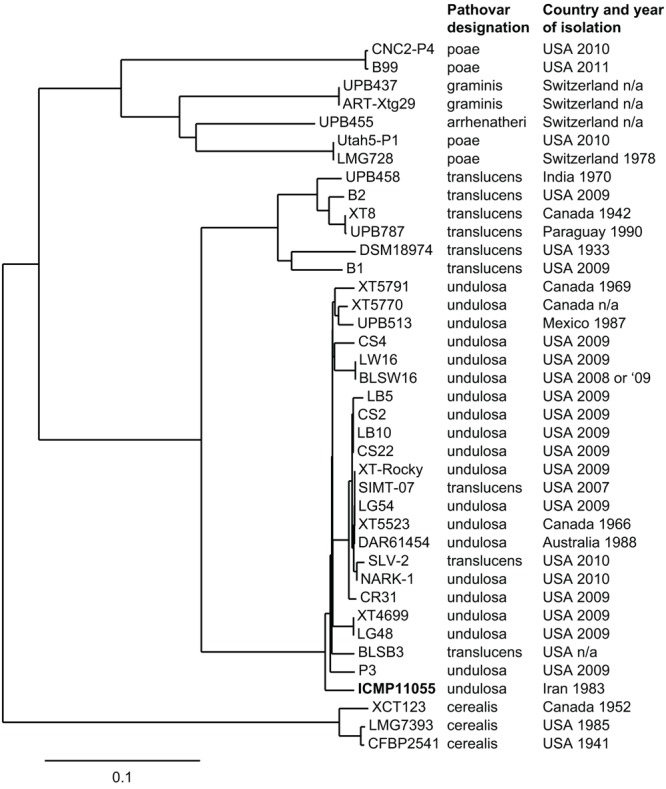
Phylogenetic tree based on whole genome SNP analysis showing the relationship of ICMP11055 to several other *X. translucens* strains. To the right, the pathovar each strain was accessioned as and the country and year of isolation are provided. The sequences used for comparison to ICMP11055 were published previously (Wichmann et al., 2013; Pesce et al., 2015; Peng et al., 2016; Langlois et al., 2017). The comparison was done using the XT4699 genome as the reference.

Genome-wide SNP comparison of ICMP11055 with the XT4699 genome and several other available *X. translucens* genome sequences (**Figure 2**) showed that, with three exceptions, strains accessioned as Xtc, Xtt, and Xtu group in distinct clades, and that within the Xtu clade, ICMP11055 resides on a branch separate from the other Xtu strains. The exceptions are three strains accessioned as Xtt that group with the Xtu strains; these are likely misnamed, Xtu strains. A fourth clade contains strains accessioned under pathovars graminis (Xtg), poae (Xtp), and arrhenatheri (Xta). With the exception of ICMP11055 and one Australian Xtu strain, DAR61454, all the strains in the Xtu clade are from North or Central America, and DAR61454 groups tightly with the American strains.

### Comparison of the ICMP11055 and XT4699 Genomes

**FIGURE 3:**
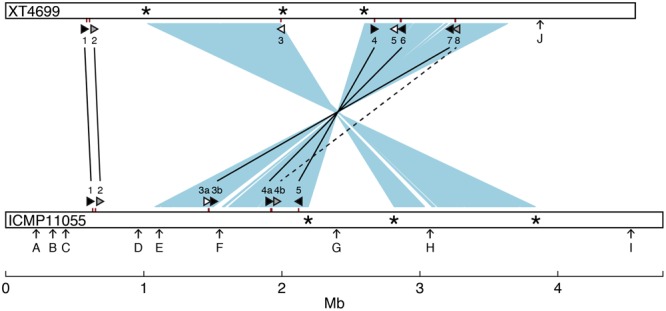
Comparison between the genomes of *X. translucens* pv. undulosa strains ICMP11055 and XT4699. The genomes are represented linearly as open bars. Two major inverted and rearranged regions are indicated by light blue shading between the genomes. Asterisks mark IS elements present at the termini of the rearranged regions. Tick marks on the genomes indicate the locations of *tal* genes. The *tal* genes are also represented, not to scale, as triangles (see **Figure 4** for RVD sequences). Perfectly or nearly perfectly conserved (1 or 0 RVD differences) *tal* genes are filled black and connected by a solid line. The *tal2* gene of ICMP11055 and the XT4699 ortholog, which diverge in the last 5 of the 18 total RVDs, are filled gray and connected by a solid line. Tal4b of ICMP11055 and Tal8 of XT4699, which show partial RVD sequence similarity, are filled gray and connected by a dashed line. The remaining *tal* genes, unique to each strain, are filled white. Arrows labeled with a capital letter mark genomic regions unique to one or the other strain, as follows: A 10 kb genomic region, encoding putative VGR related proteins for Type VI secretion; B, 14 kb genomic region comprising Types II and IV secretion system genes; C, 15 kb region, encoding a CRISPR gene cluster; D, 12 kb region, harboring phage related genes; E 11 kb region, harboring coding sequences for cyclolysin secretion ATP-binding protein, hemolysin secretion protein D, and alkaline phosphatase; F, containing putative VGR related protein genes for Type VI secretion and phage related genes; G, 15 kb region, encoding phage related proteins; H, 54 kb region, encoding phage related proteins; I, 15 kb region, encoding putative VGR related proteins for Type VI secretion; J, 10 kb region, with genes for type III restriction and modification systems.

A comparison of general features and selected gene content of the ICMP11055 and XT4699 genomes is presented in **Table 1**. A schematic alignment is shown in **Figure 3**. The ICMP11055 genome is 200 kb larger than that of XT4699. Nine extra genomic regions (>10 kb) are present in ICMP11055 (**Figure 3**). These include regions containing prophage elements (Regions D, F, G, H), a CRISPR gene cluster (Region C), VGR (valine-glycine repeats)-related genes of the Type VI secretion system (Regions A, F, I), genes encoding cyclolysin secretion ATP-binding protein, hemolysin secretion protein D and alkaline phosphatase (Region E), and a gene cluster for Type II and Type IV secretion systems (Region B). Content in each of these regions is unique to ICMP11055 except the VGR-related genes (Regions A, F, I): VGR-related genes are present in other regions shared by ICMP11055 and XT4699. Only one genomic region (>10 kb) is unique to XT4699, being a gene cluster predicted to encode a type III restriction and modification system (Region J). IS elements are frequently found at the ends of the extra genomic regions in ICMP11055, implicating possible involvement of transposons in the acquisition or loss of these regions. Similarly, IS elements are found at the ends of two rearranged regions in the alignment between the two genomes (**Figure 3**), indicating that the rearrangements might have been mediated by transposon-related sequences. ICMP11055 and XT4699 contain seven and eight *tal* genes, respectively. Based on RVD sequence, four of these are perfectly or near perfectly conserved between the two strains, and a fifth is conserved except in the last five RVDs of its CRR (**Figure 3**).

**Table 1:** Genome content comparison between *Xanthomonas translucens* pv. undulosa strains ICMP11055 and XT4699, isolated from Kerman, Iran, and Kansas, United States, respectively.

General features	ICMP11055	XT4699
Genome size (bp)	4,761,583	4,561,137
GC content (%)	67.8	68.1
Number of predicted coding genes	3,656	3,528
TAL effector genes	7	8
Non-TAL T3E genes	32	32
rRNA operons	2	2
tRNA genes	54	54
CRISPR array	1	Not detected
Insertion sequence elements (complete/partial)	83/58	74/56

### Exopolysaccharide Biosynthesis and Type III Secretion System Genes

Xanthan, an exopolysaccharide characteristic of *Xanthomonas* spp., has been implicated in *Xanthomonas* pathogenicity or virulence in several studies (Büttner and Bonas, 2010). Xanthan biosynthesis is directed by several genetic loci including the highly conserved *gum* gene cluster, *gumB* to *gumM*. (Katzen et al., 1998; Vojnov et al., 1998). This cluster is present in ICMP11055, however, *gumG* is missing. The *gumG* gene is missing from the cluster in all other available *X. translucens* genomes as well. The missing gene was reported to be *gumF* in Xtc strain ART-Xtg29 Xtg29 (Wichmann et al., 2013), however, based on sequence alignment, *gumF* is present and *gumG* absent in that assembly (not shown). The *gumO* and *gumP* genes, which are present in group II *Xanthomonas* that infect eudicots but absent from those that infect monocots (Lu et al., 2008; Jacobs et al., 2015), are also missing from ICMP11055 and each of the other *X. translucens* strains examined.

The type III secretion system (T3SS) is key to the pathogenicity of *Xanthomonas* spp. with the notable exceptions of *X. albilineans* (Pieretti et al., 2009) and *X. cannabis* (Jacobs et al., 2015). A strain of *X. sacchari* associated with banana plants was found that lacks the T3SS but its pathogenicity was not tested (Studholme et al., 2011). The T3SS is encoded by a chromosomal cluster of more than 20 *hrp* (HR and pathogenicity) genes in several operons (Büttner and Bonas, 2010). The cluster is, as expected, present in ICMP11055. However, in contrast to many studied *Xanthomonas* strains, in ICMP11055, the *hrpG* and *hrpX* genes, which encode regulators of the T3SS, are located inside the *hrp* gene cluster (Supplementary Figure S4). This characteristic of ICMP11055 is shared by XT4699 (Peng et al., 2016) and by Xtg ART-Xtg29 (Wichmann et al., 2013).

### Repertoire of T3SS Effectors of ICMP11055

The T3SS delivers a suite of proteins called effectors into plant cells. These type III effectors (T3E) act as virulence factors by targeting host metabolic, defense, or other components to promote disease or, depending on the host genotype, as avirulence factors by triggering plant defense. A typical *Xanthomonas* genome may encode 2–30 (non-TALE) T3E (Studholme et al., 2010). Candidate T3E of ICMP11055 were identified and compared with those of XT4699 and a third Xtu strain, XT-Rocky, as well as one strain each of Xtc, Xtt, Xtg, Xtp, and Xta (CFBP 2541, DSM 18974, ART-Xtg29, B99, and UPB455, respectively; Supplementary Table S3). Thirty-two putative T3E genes were predicted from the ICMP11055 genome sequence, Among these are ten core T3E genes, including *avrBs2, xopF, xopK, xopL, xopN, xopP, xopQ, xopR, xopX, and xopZ*, that were previously reported present in the genomes of all examined *Xanthomonas*^1^ except *X. albilineans, X. cannabis, X. sacchari*, and *X. campestris* pv. armoraciae, the last of which carries only *xopP* and *xopR*. Each is present also in the other *X. translucens* genomes examined, except *xopK*, which is disrupted by a frameshift mutation in the Xtg, Xtp, and Xta strains, and *xopR*, which is frameshifted in the Xtc strain. All the strains have two copies of *xopF*, though one copy is disrupted by a frameshift in the Xtp and Xtu strains. Each also has multiple copies of *xopX*. The *avrBs2* gene is present in two copies in each strain except the Xtg and Xtp strains, in which it is single-copy. The *xopL* and *xopP* genes are dual-copy in each of the Xtu strains, and their copy number ranges from one to four across the representative strains of the other pathovars. This copy number variation contrasts with group II *Xanthomonas* genomes, in which most of the core T3Es are present in single copy^1^. Seven other T3E genes found in ICMP11055, *hpaH* (*hopP1*), *xopB, xopC2, xopG, xopV, xopY*, and *xopAM*, are each also present in all of the other *X. translucens* strains examined, all single-copy. Four more are present in each of the three Xtu strains and all but one of the other strains: *xopAA* is present in all but the Xtg strain, at single copy; *xopAD* is present in each strain at single copy but is frameshifted in the Xtp strain; *xopAF* is single copy in ICMP11055 and one or two copies in the other strains except the Xta strain, from which it is absent; and *xopAP* is present at single copy in all strains but is frameshifted in the Xtt strain. ICMP11055, the two other Xtu strains, and the Xtt strain each also carries *xopAH*, which is missing from the other strains, and *xopAK*, which is also present in the Xtg strain but not in the others. The Xtu strains and the Xtc strain each carry two members of the *xopE* family, *xopE1* and *xopE5*, each at single copy. Other members of the family, *xopE2, xopE3*, and *xopE4*, are distributed unevenly across the strains representing other pathovars, at single copy where they occur. Finally, four T3E not found in ICMP11055, *avrBs1, xopI, xopJ1*, and *xopJ1*, are present in one or more of the other strains examined. Overall, there is substantial variation in T3E content across the pathovar representatives examined, however, with the exception of the presence of *xopJ1* in XTU-Rocky, T3E content is identical across ICMP11055 and the other Xtu strains.

### TALEs of ICMP11055

**FIGURE 4:**
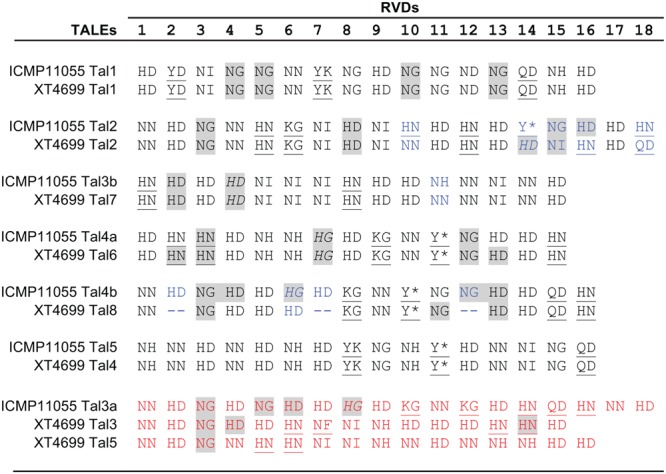
Alignment of repeat-variable di-residues (RVDs) of TALEs of *X. translucens* pv. undulosa strains ICMP11055 and XT4699. RVDs that differ between otherwise highly conserved TALEs are shown in blue font. RVDs of TALEs unique to one or the other strain are shown in red font. An asterisk indicates that the second aa of the RVD is missing. Unusual RVDs discussed in the text are underlined. RVDs in a repeat of the 34 aa type, missing the fourth reside from the end relative to the 35 aa type, are highlighted in gray; RVDs in italics represent repeats of the 34 aa type that end in the sequence Q-DHG, in which the hyphen indicates the missing residue relative to the 35 aa repeat type. The other repeats of the 34 aa type end in Q-ECG.

Many members of the genus *Xanthomonas* harbor one or more *tal* genes, and individual strains in some species may harbor several *tal* genes (up to 29 *tal* genes have been observed in individual *X. oryzae* pv. oryzicola strains) (Bonas et al., 1989; Yang and White, 2004; Boch and Bonas, 2010; Wilkins et al., 2015). In the comparison of T3E content described above, presence of *tal* genes, or in the draft genomes evidence thereof, was also examined (Supplementary Table S3). Similar to ICMP11055 and XT4699, the third Xtu strain, XTU-Rocky, harbors at least seven *tal* genes. The Xtc strain (CFBP 2541) carries two. Partial sequences were detected in the draft genome assemblies for the Xtt, Xtp, and Xta strains. None was found in the Xtg sequence. The seven ICMP11055 *tal* genes encode proteins with 15 to 18 RVDs. The TALEs of ICMP11055 are partially distinct from those of XT4699 in their DNA binding specificities (RVD sequences) (**Figure 4**). Four are perfectly or nearly perfectly conserved (1 or zero differences in RVDs) between the two strains. One ICMP11055 TALE, Tal2, shares the first 13 RVDs with Tal2 of XT4699, but the two TALEs diverge in the remaining five RVDs. There is no obvious similarity among the remaining two TALEs of ICMP11055 and three TALEs of XT4699.

The TALEs of ICMP11055, like those of XT4699 (Peng et al., 2016), show unusual RVD composition. Across all TALEs, HD, NG, NI, and NN are the most common RVDs; several others occur less frequently (Moscou and Bogdanove, 2009; Boch and Bonas, 2010; Rinaldi et al., 2017). In addition to the common RVDs, ICMP11055 and XT4699 TALEs harbor some RVDs so far not observed in TALEs in other *Xanthomonas* species, including NF, KG, QD, Y^∗^, YD, and YK (**Figure 4**). Also distinct from TALEs in other *Xanthomonas* species, HN is relatively frequent in ICMP11055 and XT4699 TALEs, as it is in *Ralstonia* RipTALs (Lange et al., 2013). On the other hand, the RVD NK, which occurs at higher frequencies in *Ralstonia* than in *Xanthomonas*, is not found in the ICMP11055 or XT4699 TALEs. The TALEs encoded by the few genes individually cloned from other Xtu strains (Peng et al., 2016) align well with ICMP11055 and XT4699 TALEs, while the two available Xtc CFBP 2541 TALE sequences (Pesce et al., 2015) and six of the eight identified Xtt DSM 18974 TALE sequences (Jaenicke et al., 2016) are unique (Supplementary Figure S5). One of the remaining Xtt DSM 18974 TALEs aligns well with ICMP11055 Tal2 up to RVD 14 but diverges thereafter, distinctly from XT4699 Tal2. The other aligns well with ICMP11055 Tal3b (and XT4699 Tal7). The Xtc CFBP 2541 TALEs contain the RVDs KI and GI, not found in any of the other TALEs. The CFBP 2541 TALEs and one of the DSM 18974 TALEs also contain an NK. Of all the TALEs, only one, from CFBP 2541, contains an NS, an RVD that is generally common in *Xanthomonas* TALEs but not yet found in RipTALs. Thus RVD composition appears to set *X. translucens* TALEs apart both from TALEs of group II *Xanthomonas* species and from RipTALs.

**FIGURE 5:**
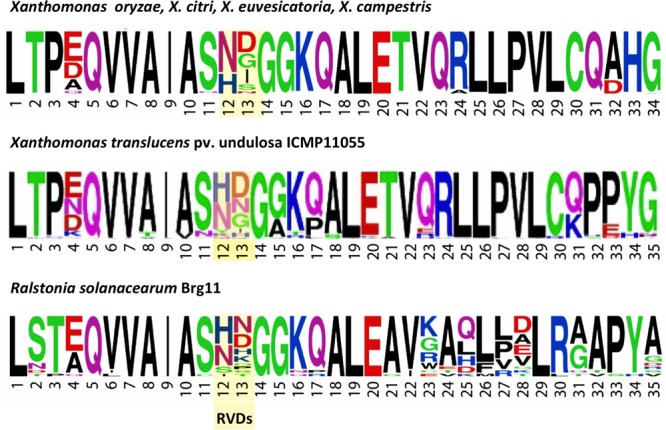
Amino acid sequence motifs of TALEs of *X. translucens* pv. undulosa strain ICMP11055 relative to TALEs of other *Xanthomonas* species and a RipTAL of *Ralstonia solanacearum*. WebLogos (Crooks et al., 2004) illustrate the frequency of individual amino acids (by letter height, using the single letter code) at each position across the repeats of TALEs from representative strains of (top) *X. oryzae, X. citri, X. euvesicatoria, and X. campestris*, (middle) the TALEs of *X. translucens* pv. undulosa ICMP11055, and (bottom) RipTAL Brg11 of *Ralstonia solanacearum*. The RVD positions are highlighted. The WebLogo for the ICMP11055 TALEs was generated in this study. The others were published previously (Schornack et al., 2013) and are used with permission of Annual Reviews; permission conveyed through Copyright Clearance Center, Inc.

Transcription activator-like effector repeats are typically 34 amino acids in length, or 33 when the RVD is missing a residue. An exception is Hax2 of *X. campestris* pv. armoraciae strain 5, which consists solely of 35 aa repeats; Hax3 and Hax4 from that strain, however, contain only the more typical 34 aa repeat type (Kay et al., 2005). AvrHah1 from *X. gardneri* comprises both 34 and 35 aa repeat types (Schornack et al., 2008). *Ralstonia* RipTAL repeats are 35 amino acids long (Bogdanove et al., 2010). With the exception of ICMP11055 Tal5 and its ortholog in XT4699, which contain exclusively repeats of the 35 aa type, the ICMP11055 and XT4699 TALEs (and the other *X. translucens* TALEs for which sequence is available) each comprise both 34 and 35 aa repeat types. Interestingly, although Tal4a in ICMP11055 and Tal6 in XT4699 have identical RVD sequences, they differ at two repeats in the repeat length, 34 vs. 35 aa. As in the RipTALs, the extra amino acid in the 35 aa repeats of the *X. translucens* TALEs is at position 32 or 33, within the inter-repeat loop (Mak et al., 2012). And, whereas the penultimate two residues of the repeat consensus for TALEs of *X. oryzae, X. citri, X. euvesicatoria*, and *X. campestris* are HG, they are PY in the repeat consensus for ICMP11055, as in the repeat consensus of a typical RipTAL (**Figure 5**). However, like other *Xanthomonas* TALEs and in contrast to RipTALs, the non-RVD, backbone residues of the ICMP11055 TALE repeats are relatively invariable. Thus, the overall *X. translucens* TALE repeat structure appears to be somewhat intermediate between that of RipTALs and that of TALEs of group II *Xanthomonas* species. The N- and C- terminal domains outside the repeats, however, are typical of TALEs in group II *Xanthomonas* species (Supplementary Figure S6).

The *tal5* gene of ICMP11055, in addition to being the only ICMP11055 *tal* gene comprising exclusively repeats of the 35 aa type, has a promoter sequence distinct from the other ICMP11055 *tal* genes (not shown), and the encoded N- and C-terminal regions also differ (compare Tal5 and the representative Tal3a in Supplementary Figure S6). These differences suggest that *tal5* was acquired through horizontal gene transfer sometime after the other *tal* genes were established in the lineage that gave rise to ICMP11055, and that those other *tal* genes had arisen through duplication of a distinct, ancestral *tal* gene.

### Contributions of *tal2* and *tal4b* to the Virulence of ICMP11055

**FIGURE 6:**
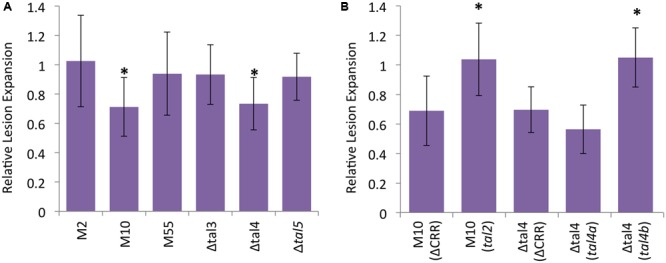
Contributions of *X. translucens* pv. undulosa strain ICMP11055 Tal2 and Tal4b to virulence. **(A)** Lengths of lesions caused by *tal* gene knockout or deletion mutant strains relative to the wild type, inoculated side-by-side to leaves of 3-week old *Triticum aestivum* cv. Chinese Spring plants. Mutant strains include *tal1* knockout M2, *tal2* knockout M10, *tal3a* knockout M55, *tal3a* and *tal3b* deletion mutant Δtal3, *tal4a* and *tal4b* deletion mutant Δtal4, and *tal5* deletion mutant Δ*tal5*. Values represent relative expansion of lesions beyond the inoculated area measured 9 days after inoculation (see Supplementary Figure S7 for images of representative leaves). At least twelve, paired inoculations were measured for each strain. An asterisk indicates a significant difference (*p* < 0.01) from the wild type lesion measurement. Error bars represent standard deviation. **(B)** Genetic complementation of M10 by a plasmid-borne, dTALE equivalent of *tal2* and of Δtal4 by a *tal4b* clone but not a *tal4a* clone (see Materials and Methods and Supplementary Table S1); ΔCRR, control construct lacking the CRR (Supplementary Table S1). Assays were carried out as in **(A)**, and values relative to the wild type are presented, but here an asterisk indicates a significant difference from the relative value for the corresponding mutant transformed with the control construct. The experiments in **(A,B)** were repeated at least twice and yielded similar results in each case.

To determine whether any of the ICMP11055 TALEs contribute to virulence, *tal* gene knockout mutant derivatives of ICMP11055 were generated. The mutant strains include single-crossover, knockout mutants M2 (*tal1*), M10 (*tal2*), and M55 (*tal3a*) and deletion mutants Δtal3 (*tal3a* and *tal3b*), Δtal4 (*tal4a* and *tal*4b), and *Δtal5* (*tal5*) (see Materials and Methods). The mutants were inoculated to wheat cv. Chinese Spring leaves, and the lengths of resulting lesions were compared to those caused by the wild type strain at 9 days after inoculation. M10 and Δtal4 caused shorter lesions than the wild type strain, and the lengths of the lesions produced by the other mutants were not significantly different from those of the wild type strain (**Figure 6A** and Supplementary Figure S7). A dTALE equivalent of *tal2* (see Materials and Methods) introduced on a plasmid into M10 restored full virulence (**Figure 6B**). Cloned *tal4b*, but not *tal4a*, likewise restored full virulence to Δtal4 (**Figure 6B**). These results indicate that *tal2* and *tal4b* encode major TALEs that contribute non-redundantly to the virulence of ICMP11055.

To detect virulence contributions of any ICMP11055 TALEs that might function redundantly, we next attempted a gain-of-function approach by expressing each ICMP11055 *tal* gene individually in the Xtc strain CFBP 2541, which is weakly virulent on Chinese Spring. In Chinese Spring leaves, watersoaking caused by CFBP 2541 on Chinese Spring leaves remains limited to the infiltrated area, in contrast to the spreading watersoaking caused by ICMP11055. None of the ICMP11055 *tal* genes, however, changed the extent of watersoaking caused by CFBP 2541, including *tal2* and *tal4b* (data not shown).

## Discussion

In this study, the genome of Xtu ICMP11055, a highly pathogenic strain isolated in Iran, was sequenced using SMRT technology and compared with the complete genome of another strain of Xtu, XT4699, isolated from United States, as well as draft or complete genomes of several other *X. translucens* strains. Variation across strains in *gum* and T3SS genes was examined, and T3E content was compared. An analysis of TALEs encoded in the ICMP11055 genome and other available *X. translucens* sequences was carried out, and the ICMP11055 TALEs were functionally characterized by mutation and by heterologous expression.

The ICMP11055 and XT4699 genomes differ slightly in size and show two major rearrangements relative to one another, but overall are highly similar. Whole genome SNP analysis revealed a high degree of homogeneity overall among a moderate collection of Xtu genomes representing strains from North and Central America, a strain from Australia, and the Iranian strain ICMP11055, but differentiated ICMP11055 from the others. The American strains are diverse with respect to place and year isolated. Their overall similarity is striking. Notably, except for the three, likely misnamed Xtt strains that grouped with the Xtu strains, all of the Xtu strains grouped uniquely together, apart from a group of exclusively Xtt strains, a group of exclusively Xtc strains, and a fourth group of strains designated as pv. Xtg, Xtp, or Xta. This result suggests that Xtu, defined by its host specificity, is a robust biological entity representing strains with a common genetic basis for that host specificity. The overall grouping and the distinct position of ICMP11055 among the Xtu strains suggest that future comparison of a larger collection of geographically diverse Xtu, Xtc, and Xtt strains would help narrow a list of group-specific polymorphisms that could be examined for candidate determinants of host specificity.

Comparison of the *gum* gene content and the arrangement of T3SS genes across representative *X. translucens* strains revealed no polymorphism correlated with host range. A role for xanthan in pathogenicity of *X. translucens* strains has not been reported, but *gum* genes have been found to be important for many other *Xanthomonas* species and pathovars (Büttner and Bonas, 2010). The absence of *gumG* from the *X. translucens* genomes contrasts with type II *Xanthomonas*. Across group II *Xanthomonas*, however, *gumG* is the most divergent of the genes in the cluster and shows roughly 40% identity to *gumF*, suggesting that it arose from duplication of *gumF* (Lu et al., 2008). The *X. translucens* cluster may represent the more ancestral, since *gumG* is also missing from another member of the Xanthomonadaceae, *Xylella fastidiosa* (Simpson et al., 2000). The absence of *gumO* and *gumP* from ICMP11055 and the representative genomes of the five other *X. translucens* pathovars examined also resembles *Xylella fastidiosa*. These genes, which encode 3-axoacyl-(acyl carrier protein) synthase and a metal-dependent hydrolase, respectively, appear not to be critical for xanthan biosynthesis, although their absence may restrict the spectrum of precursor compounds that could be used. That they are missing from the *X. translucens* strains, each of which is a monocot pathogen, is consistent with their absence from group II *Xanthomonas* pathogenic to monocots (and presence in eudicot pathogenic *Xanthomonas*) and may indicate a role in host specificity at that level.

Several studies have examined the role of the T3SS in *X. translucens*. T3SS mutants of ART-Xtg29 caused considerably less severe symptoms in ryegrass (*Secale cereale*) compared to the wild type strain, yet they did infect the plants. Moreover, *in planta* multiplication of these mutants was not reduced significantly (Wichmann et al., 2013). Mutation of *hrcT* resulted in a decrease in severity of symptoms caused by Xtt strain UPB886 in barley but, similar to mutation of the T3SS in ART-Xtg29, did not lead to complete loss of virulence in this host plant either (Pesce et al., 2015). Mutation of *hrcC* in XT4699 resulted in complete loss of water-soaking on wheat (Peng et al., 2016). Based on our results demonstrating the importance of Tal2 and Tal4b of ICMP11055 for lesion expansion, discussed below, the T3SS is clearly important to symptomogenesis by ICMP11055 as well. Whether the T3SS plays a critical role in proliferation of Xtu *in planta* remains to be assessed.

The T3SS gene cluster in ICMP11055 and other *X. translucens* strains was found to differ from that of most *Xanthomonas* by containing the regulatory genes *hrpG* and *hrpX* within it, rather than at a distinct chromosomal location. In this respect, the cluster resembles that of *R. solanacearum* (the orthologous genes in this species are designated *hrpG* and *hrpB*, respectively; Salanoubat et al., 2002). The *hrpG* and *hrpX* genes are not found in the handful of *Xanthomonas* species known to lack the T3SS.

Several of the *Xanthomonas* non-TALE T3E represented in ICMP11055 or their orthologs in other plant pathogenic genera are known to play important roles in host–pathogen interactions. Among the core T3Es, AvrBs2 is one of the first effectors shown to contribute to virulence. The protein possesses a conserved glycerolphosphodiesterase domain suggested to play roles in both osmotic adaptation and plant host signaling (Swords et al., 1996). Another core effector, XopR, is required for full virulence of *X. oryzae* pv. oryzae strain 13571, but its mode of action is not yet understood (Zhao et al., 2013). Core effector XopZ potentially interferes with host innate immunity. The homolog of XopZ in *Pseudomonas syringae* pathovars, HopZ1, induces transcription of jasmonate-dependent genes and represses salicylate-dependent defense responses against bacteria (Furutani et al., 2009; Song and Yang, 2010; Sinha et al., 2013; Teper et al., 2015). In *X. axonopodis* pv. manihotis, AvrBs2, XopX, and XopZ have been shown to be required for full virulence, core effectors XopN and XopQ to contribute redundantly to virulence, and AvrBs2 and XopR to suppress pathogen-associated molecular pattern-triggered immunity (Medina et al., 2017). Among the non-core ICMP11055 T3Es present in most of the *X. translucens* strains compared, XopAP, which was shown in *X. euvesicatoria* strain 85-10 to contribute to virulence, enhances jasmonic acid (JA) production to activate JA-induced defenses and repress salicylic acid-induced defense reactions (Teper et al., 2015). And, XopAF, which is present also in *X. axonopodis* pv. vesicatoria 91–118 and *X. oryzae* pv. oryzicola BLS256, is a homolog of the *P. syringae* effector HopAF1, which suppresses plant basal immunity (Astua-Monge et al., 2000; Gibly et al., 2004; Balaji et al., 2007; Bogdanove et al., 2011). One of the two T3Es common to the Xtu strains and the Xtt strain but missing in all or most of the others, XopAK, is a homolog of *P. syringae* effector HopK1, which localizes to the chloroplast, induces jasmonic acid-responsive genes, and suppresses host immune responses (He et al., 2004; Jamir et al., 2004; Li et al., 2014).

The comparison of T3E content of representative strains revealed substantial variation across *X. translucens* pathovars, not only in presence or absence of particular effectors, but also in copy number of some effectors. The extent to which the non-core effectors function in an overlapping or redundant fashion, and whether any contributes to host specificity of *X. translucens* pathovars are unknown. Yet, the variability in T3E content is a salient feature of *X. translucens*. Determining the functional relevance of this variability, particularly with regard to host specificity, is a potentially useful goal for future work. At the same time, given the variability observed across pathovars, the near identity of T3E content of the geographically and temporally distinct Xtu strain ICMP11055, isolated in 1983, to that of the two American Xtu strains, isolated 26 years later, is striking. Similar to the grouping by whole genome SNP analysis, this result supports Xtu as a robust taxonomic unit, and lends weight to the notion that its non-core T3E content contributes to its host specificity.

The TALEs of ICMP11055, and other *X. translucens* strains, based on the available sequences appear to constitute a divergent sub-family, similar overall to TALEs of group II *Xanthomonas* species but sharing some features of the RipTALs of *Ralstonia*. The N- and C- terminal domains are typical of TALEs in group II *Xanthomonas* species, but RVD composition sets the *X. translucens* TALEs apart, and their repeat structure is somewhat intermediate between the group II TALEs and the RipTALs. Whether the features shared with RipTALs result from convergent evolution, relatively closer relatedness of *X. translucens* TALEs than group II TALEs to RipTALs, or an ancient recombination event between TALE and RipTAL progenitors is unclear. The aligned nucleotide sequences of representative *X. translucens* TALEs (Tal3a and Tal5 of ICMP11055) and a typical RipTAL (RipTALI-1; Schandry et al., 2016) do not help distinguish among these possibilities, as no significant similarity is observed (not shown).

Comparison among the ICMP11055 *tal* genes revealed that *tal5* is distinct in several respects from the others, suggesting independent acquisition in the lineage that gave rise to the strain. The gene is identical to *tal4* in XT4699, suggesting that it was acquired prior to divergence that led to the two strains. A *tal5* ortholog has not been identified among the small number of available *tal* gene sequences for other *X. translucens* strains (Supplementary Figure S5). Based on the greater similarity of the N- and C- terminal sequences of Tal5 to those of Tal3a (of ICMP11055) than to TALEs of group II *Xanthomonas* species, and its harboring the RVDs QD, YK, and Y^∗^, we speculate that the gene, or an ancestor, was introduced from a strain more closely related to Xtu than to the *Xanthomonas* species in group II.

The *tal5* gene is one of only four *tal* genes shared by ICMP11055 and XT4699, out of their seven and eight respective totals. This relative lack of conservation contrasts with the near identity of the non-TALE T3E content of these two strains. Mutational analysis of the ICMP11055 *tal* genes revealed Tal2 and Tal4b to be important virulence factors in wheat (cv. Chinese Spring). Neither of these is among the TALEs shared by XT4699. A Tal2 apparent ortholog exists in XT4699, but it differs in the last five out of 18 RVDs, and only a TALE with partial, fragmented RVD sequence similarity to Tal4b can be found in XT4699. This divergence does not preclude the possibility that Tal2 and the XT4699 ortholog share the same DNA target, given the degeneracy of the TALE-DNA binding code (Moscou and Bogdanove, 2009) and the tolerance of TALEs to DNA sequence mismatches at the 5′/C-terminal end (Meckler et al., 2013). Likewise, the XT4699 TALE with limited similarity to Tal4b, or some other XT4699 TALE, might target the same gene as Tal4b at a different location in the promoter, or target a different but functionally equivalent gene. The lack of perfect conservation of the two ICMP11055 virulence factors in XT4699 is nonetheless intriguing, hinting that TALEs might be a focal point for Xtu adaptation to different host genotypes or possibly even to different homeoalleles of important *S* genes within a polyploid host. Along the same lines, the four ICMP11055 TALEs that did not demonstrably contribute to the virulence of the strain toward the hexaploid wheat cultivar Chinese Spring might function redundantly by targeting the same *S* gene or different *S* gene homeoalleles in that cultivar, or they may be important in a different wheat genotype or a different host species. In bacterial blight of cassava, TAL20 from *X. axonopodis* pv. manihotis 668 increased watersoaking when expressed in the less virulent strain CIO151 (Castiblanco et al., 2013; Cohn et al., 2014). Thus, to control for redundancy, we tested each of the four ICMP11055 TALEs for which knockout yielded no phenotype by introducing them into Xtc strain CFBP 2541 and assaying for gain-of-function. None increased the virulence of that strain, However, neither did Tal2 or Tal4b. Thus, further characterization in both loss of function experiments and gain of function experiments (using an appropriate, low-virulence Xtu strain) in different hosts will be necessary to rule out a role in disease for those four ICMP11055 TALEs. Of course, as suggested previously (Bogdanove et al., 2010), they may have no important direct function, conferring selective advantage only indirectly as coding sequence for rapid adaptation via recombination.

The question of possible roles for the other ICMP11055 TALEs notwithstanding, with the discovery that Tal2 and Tal4b contribute to lesion expansion, Xtu ICMP11055 joins a growing list of *Xanthomonas* strains that have been shown to depend on one or more TALEs for full virulence. As described in the introduction, direct targets of TALEs important for disease development include genes encoding sugar exporters, transcription factors, and a putative sulfate transporter, and a pectate lyase gene has been identified as an important secondary target, induced by a TALE-activated transcription factor. Future work to identify the relevant targets of Tal2 and Tal4b will reveal whether homologs of any of these, or new types of *S* genes, are involved in bacterial leaf streak of wheat. The complete RVD sequences, the mutant strains, and the clones of Tal2 and Tal4b described above open the door to identifying those *S* genes. Using the available draft genome of wheat^2^, candidates can be isolated that are activated by these effectors and that harbor matching EBEs in their promoters, and then tested using dTALEs (Cernadas et al., 2014). Given the relatively large contributions to virulence by Tal2 and Tal4b, incorporation of alleles of the corresponding susceptibility genes that lack the EBE could be expected to provide a practical level of resistance to ICMP11055 and other strains that depend on Tal2 and Tal4b or equivalent TALEs. Such alleles might be obtained through a targeted search of native germplasm (Hutin et al., 2015) or generated by targeted EBE mutagenesis using genome editing (Li et al., 2012; Wang et al., 2014).

## Author Contributions

NFC and AB conceived and designed the study, with assistance from ZP, SL, MS-B, and FW. NFC carried out the experiments, with assistance from LW. HR provided essential materials. NB assembled the ICMP11055 genome. NFC, ZP, ZL, FW, and AB analyzed and interpreted the data. NFC, NB, and AB prepared the paper, with assistance from ZP, SL, ZL, and FW.

## Conflict of Interest Statement

The authors declare that the research was conducted in the absence of any commercial or financial relationships that could be construed as a potential conflict of interest.

## Footnotes

1 http://www.xanthomonas.org

2 www.wheatgenome.org
