# Supplementary material for: Complete Genome Sequencing and Targeted Mutagenesis Reveal Virulence Contributions of Tal2 and Tal4b of Xanthomonas translucens pv. undulosa ICMP11055 in Bacterial Leaf Streak of Wheat
Source: Front Microbiol. 2017 Aug 10;8:1488. doi: 10.3389/fmicb.2017.01488 (PMC5554336; doi:10.3389/fmicb.2017.01488)
Supplement: Supplementary file 1 [file Presentation_1.PDF]

Supplementary Material

Complete genome sequencing and targeted mutagenesis reveal virulence contributions of Tal2 and Tal4b of *Xanthomonas translucens* pv. undulosa ICMP11055 in bacterial leaf streak of wheat

Nargues Falahi Charkhabi[1,2], Nicholas J. Booher[1], Zhao Peng[3,4], Li Wang[1], Heshmat Rahimian[5], Masoud Shams-bakhsh[2], Zhaohui Liu[6], Sanzhen Liu[3], Frank F. White[3,4], and Adam J. Bogdanove[1]*

[1] Plant Pathology and Plant-Microbe Biology Section, School of Integrative Plant Science, Cornell University, Ithaca NY USA.

[2] Plant Pathology Department, Tarbiat Modares University, Tehran, Iran.

[3] Department of Plant Pathology, Kansas State University, Manhattan, KS USA.

[4] Department of Plant Pathology, University of Florida, Gainesville, FL USA.

[5] Department of Plant Protection, Sari University of Agriculture Science and Natural Resources, Sari, Mazandaran, Iran.

[6] Department of Plant Pathology, North Dakota State University, Fargo, ND, USA.

*** Correspondence:** Adam J. Bogdanove, Plant Pathology and Plant-Microbe Biology Section, School of Integrative Plant Science, Cornell University, 334 Plant Science Building, Ithaca, NY, 14853, USA.

ajb7@cornell.edu

Supplementary Tables

Table S1. Bacterial strains and plasmids used.

Strain or plasmid	Source	More information
Xanthomonas translucens pv. cerealis CFBP 2541	D. W. Dye	Isolated from *Bromus inermis*, USA, 1941
Xanthomonas translucens pv. undulosa ICMP11055	(Alizadeh and Rahimian, 1989)	Isolated from *Triticum aestivum*, Iran, 1983
Xtu ICMP11055-M2, Km^r	This study	ICMP11055 marker exchange mutant with disruptive insertion mapping to *tal1*
Xtu ICMP11055-M10, Km^r	This study	ICMP11055 marker exchange mutant with disruptive insertion mapping to *tal2*
Xtu ICMP11055-M55, Km^r	This study	ICMP11055 marker exchange mutant with disruptive insertion mapping to *tal3b*
Xtu ICMP11055 Δtal3	This study	ICMP11055 unmarked deletion mutant of *tal3a* and *tal3b*
Xtu ICMP11055 Δtal4	This study	ICMP11055 unmarked deletion mutant of *tal4a* and *tal4b*
Xtu ICMP11055*Δtal5*, Cm^r	This study	ICMP11055 marked deletion mutant of *tal5*
pK18*mobsacB*	(Kvitko and Collmer, 2011)	Km^r
pQD5	This study	Derivative of pHD5 (Cermak et al., 2011) mutated using primers 1658 and 1659 to encode RVD 'QD,' Tc^r
pQD-LR	This study	Analog of pHD-LR (Cermak et al., 2011) created by cloning double stranded oligonucleotide 1760[a] in pCR8/GW/TOPO.
pYN1	This study	Derivative of pNN1 (Cermak et al., 2011) mutated using primers 1662 and 1663 to encode RVD 'YN,' Tc^r
pY*1	This study	Derivative of pYN1 mutated using primers 1712 and 1713 to encode RVD 'Y*,' Tc^r
pYN8	This study	Derivative of pNN8 (Cermak et al., 2011) mutated using primers 1662 and 1663 to encode RVD 'YN,' Tc^r
pY*4	This study	Derivative of pNN4 (Cermak et al., 2011) mutated using primers 1714 and 1715 to encode RVD 'Y*,' Tc^r
pYK8	This study	Derivative of pYN8 mutated using primers 1716 and 1717 to encode RVD 'YK,' Tc^r

pKEB31	(Cermak et al., 2011)	pDD62 derivative containing Gateway destination vector cassette (Invitrogen) between *Xba*I and *Bam*HI sites, Tc^r
pSM7	(Makino, 2005)	pBluescriptII-KS(+) (Invitrogen) containing the 4.5 kb *Pst*I fragment of *tal* gene aB4.5 (Bai et al., 2000) interrupted at repeat nine by EZTN5*Not*IKan-3 transposon, Km^r
pAC99	(Verdier et al., 2012)	pKEB31 containing *tal1c* of BLS256 missing the *Sph*I repeat-encoding fragment, Tc^r
pTAL1	(Cermak et al., 2011)	Gateway compatible vector encoding the N- and C-terminal domains of Tal1c of BLS256 (missing the repeat-encoding *Sph*I fragment) for assembly of full-length TAL effector genes by Golden Gate cloning.
pNFC31	This study	pAC99 with the *Sph*I repeat-encoding fragment of *tal1* of ICMP11055, Tc^r
pNFC32	This study	pAC99 with the Golden Gate assembled equivalent of the *Sph*I repeat-encoding fragment of *tal2* of ICMP11055, Tc^r
pNFC33	This study	pAC99 with the Golden Gate assembled equivalent of *tal3a* of ICMP11055, Tc^r
pNFC34	This study	pAC99 with the Golden Gate assembled equivalent of *tal3b* of ICMP11055. Tc^r
pNFC35	This study	pAC99 with the *Sph*I repeat-encoding fragment of *tal4a* of ICMP11055, Tc^r
pNFC36	This study	pAC99 with the *Sph*I repeat-encoding fragment of *tal4b* of ICMP11055, Tc^r
pNFC37	This study	pAC99 with the Golden Gate assembled equivalent of *tal5* of ICMP11055, Tc^r
pdCas9	(Bikard et al., 2013)	Cm^r

[a] 5′-TAAGCTCGGGCCCGTCTCGCTATCGCCAGCCAAGATGGCGGCAAGCAAGCGCTCGAAAGCATTGTGGCCCAGCTGAGCCGGCCTGATCCGGCGTTGGCCGCGTTGACCAACGAGAGACGTCTAGACCAGCCA-3′

Table S2. Primers used.

No.	Sequence [a]	Reference
235	5´-GGAGGCCTTGCTCACGGATGC-3´	(Cernadas et al., 2014)
236	5´-GGCCGGTGACAGCACGATCCG-3´	(Cernadas et al., 2014)
395	5´-TCCCGTTGAATATGGCTCATAACACCCC-3´	(Cernadas et al., 2014)
397	5´-GTCCACCTACAACAAAGCTCTCATCAACC-3´	(Cernadas et al., 2014)
398	5´-TCCTCTTCGTTGAATGCC-3´	(Cernadas et al., 2014)
676	5´-TTGATGCCTGGCAGTTCCCT-3´	(Cermak et al., 2011)
677	5´-CGAACCGAACAGGCTTATGT-3´	(Cermak et al., 2011)
678	5´-TTGGCGTCGGCAAACAGTGG -3´	(Cermak et al., 2011)
679	5´-GGCGACGAGGTGGTCGTTGG -3´	(Cermak et al., 2011)
736	5´-TCGGCGAGCACATGGAACGG-3´	(Cermak et al., 2011)
1527	5'-AGCTTCTGGCCGGATCCCAACC -3'	This study
1571	5´-AAGGCACGGCCCATCTGGTTGC-3´	This study
1593	5´-CGAATGCATGCGGTCCCAACGTGG-3´	This study
1623	5´-TAGCGAATTCCAGACGCTGGAAATCATAG-3´	This study
1624	5´-TTTGCCCGGGGATATGAAGTTTCAATTCTG-3´	This study
1625	5´-CTCAACGCTTTGCGATCGCAGGCG-3´	This study
1626	5´-GGTGCAGAATCTGCCTCATTCGCTTCG-3´	This study
1627	5´-TTCTCCCGGGAAAAATGCTGGCGCATGC-3´	This study
1628	5´-CACCTGCTCGACGATCTCTAGAACGC-3´	This study
1629	5´-CGAAGCGTTTGACGATGAAGGTGTCC-3´	This study
1630	5´-GAAGTTGCCGGCATTCAACTCGTACTTG-3´	This study
1631	5´-AACTGGATCCGCGACAACCTGCTGTACG-3´	This study
1632	5´-TGGCTCTAGACGCTCCGACACTCATCC-3´	This study

1633	5´-ACCTCGGCGCTGGATTCGGAAGTG-3´	This study
1634	5´-CATCCTGATCGACGGCCAGGACATC-3´	This study
1635	5´-AGGCTCTAGACTTGCCCTTCACTTCGGC-3´	This study
1636	5´-AGCGAAGCTTCTGATTCGGCGCGAGAAG-3´	This study
1637	5´-CATCCTCGGCTACTACAGCCTATCCG-3´	This study
1638	5´-CATGATCCAGACCATGAACGGACTGAG-3´	This study
1658	5´-CTATCGCCAGCCAAGATGGCGGCAAGC-3´	This study
1659	5´-GCTTGCCGCCATCTTGGCTGGCGATAG-3´	This study
1662	5´-GCTATCGCCAGCTACAATGGCGGCAAGC-3'	This study
1663	5´-GCTTGCCGCCATTGTAGCTGGCGATAGC-3´	This study
1712	5´-GGCGGCAAGCAAGCGCTC-3´	This study
1713	5´-GGCGGCAAGCAAGCGCTC -3´	This study
1714	5´-TATCGCCAGCTACGGCGGCAAGCAAGCGCTC3´	This study
1715	5´-GCCACCACTTGGTCCGGG-3´	This study
1716	5´-CGCCAGCTACAAGGGCGGCAAGC-3´	This study
1717	5´-ATAGCCACCACTTGGTCCGGG-3´	This study
1877	5´-GGCAAGCCTGTTTCCGGGATCGGGC-3´	This study
1879	5´-AGCGACACTTCGTTGTAGGCCAACGAACC-3´	This study
1914 [b]	5´-cccggggatcctctagagtcgacGCGCGCTCAGCATGAAG-3´	This study
1915 [b]	5´-gccgatcaCCGAGCGCTGCTGCCAA-3´	This study
1916 [b]	5´-gcgctcggTGATCGGCACGTAAGAGGTTCCAA-3´	This study
1917 [b]	5´-gaatgactgTTACGCCCCGCCCTGCC-3´	This study
1918 [b]	5´gggcgtaaCAGTCATTCCTCTTTCAAAGAAGGCGGATAACTTGC AAAT-3´	This study
1919 [b]	5´-ttgcatgcctgcaggtcgacGCGCCATGCCGGCCGAG-3´	This study

[a] Restriction enzyme sites are underlined.

[b] Capital letters correspond to the primary amplified fragment and lower case letters correspond to the neighboring fragment for assembly by overlap extension PCR.

Table S3. **Comparison of T3Es across *Xanthomonas translucens* pv. undulosa ICMP11055, XT4699, and XT-Rocky, *X. translucens* pv. cerealis CFBP 2541, *X. translucens* pv. graminis ART- Xtg29, *X. translucens* pv. translucens DSM 18974, *X. translucens* pv. poae B99, and *X. translucens* pv. arrhenatheri UPB455.**[a]

Effector	ICMP11055[b]	XTU- Rocky[c]	XT4699[c]	CFBP 2541[d]	ART-Xtg29[e]	DSM 18974[f]	B99[g]	UPB455[g]
HpaH(HopP1)	+	+	+	+	+	+	+	+
XopB	+	+	+	+	+	+	+	+
XopC2	+	+	+	+	+	+	+	+
XopF	++	++	++	++	++	++	F,+	F,+
XopG	+	+	+	+	+	+	+	+
XopK	+	+	+	+	F	+	F	F
XopN	+	+	+	+	+	+	+	+
XopQ	+	+	+	+	+	+	+	+
XopV	+	+	+	+	+	+	+	+
XopX	+++	+++	+++	+++	+++	+++	++,F	++
XopY	+	+	+	+	+	+	+	+
XopZ	+	+	+	+	+	+	+	+
XopAA	+	+	+	+	ND	+	+	+
XopAD	+	+	+	+	+	+	F	+
XopAM	+	+	+	+	+	+	+	+
AvrBs1	ND	ND	ND	+	ND	ND	ND	ND
AvrBs2	++	++	++	++	+	++	+	++
XopE1	+	+	+	+	+	ND	+	ND
XopE2	ND	ND	ND	+	+	+	+	ND
XopE3	ND	ND	ND	ND	ND	+	ND	ND

Effector	ICMP11055[b]	XTU- Rocky[c]	XT4699[c]	CFBP 2541[d]	ART-Xtg29[e]	DSM 18974[f]	B99[g]	UPB455[g]
XopE4	ND	ND	ND	ND	+	ND	ND	ND
XopE5	+	+	+	+	ND	ND	ND	ND
XopI	ND	ND	ND	ND	+	ND	+	+
XopJ1	ND	+	ND	ND	ND	+	ND	+
XopJ2(AvrRxv)	ND	ND	ND	ND	+	ND	ND	ND
XopL	++	++	++	++++	+	++	+,F	++
XopP	++	++	++	+++	++	+++	++	+,F
XopR	+	+	+	F	+	+	+	+
XopAF (AvrXv3)	+	++	++	+	+	++	+	ND
XopAH(AvrB3)	+	+	+	ND	ND	+	ND	ND
XopAK(HopK1)	+	+	+	ND	+	+	ND	ND
XopAP	+	+	+	+	+	F	+	+
TALE	7	≥7	8	2	ND	P	P	P

[a] The number of plusses indicates the number of copies of the gene detected in the genome (except for TALEs, for which the number is given); ND, not detected; F, the gene harbors a frameshift mutation; P, *tal* gene sequences (typically 3’ or 5’ end, non-repeat sequences) present in one or more contigs in the genome assembly.
[b] This study.
[c] (Peng et al., 2016).
[d] (Pesce et al., 2015)
[e] (Wichmann et al., 2013)
[f] (Jaenicke et al., 2016)
[g] (Langlois et al., 2017).

Supplementary Figures

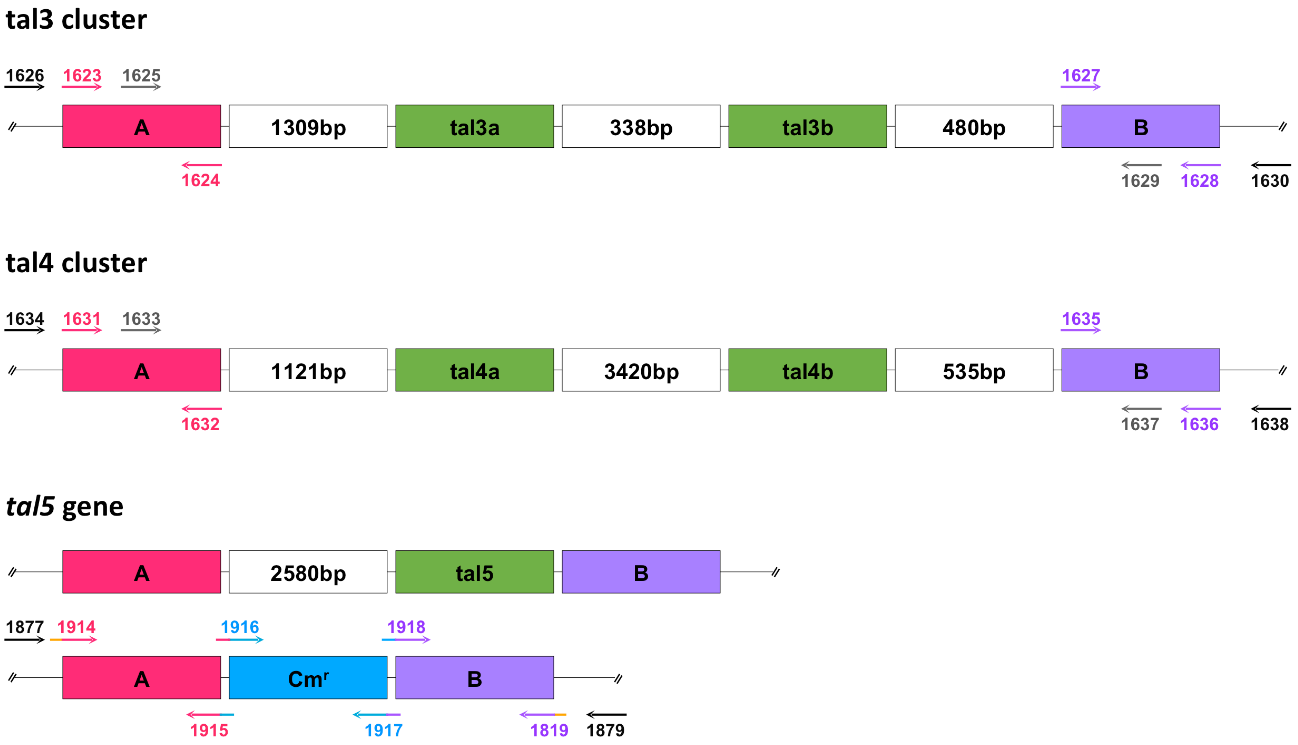

Figure S1. **Schematics of the ICMP11055 tal3 and tal4 clusters and the tal5 gene showing the respective flanking regions (A and B in each schematic) and primers used in deletion mutagenesis.** In each case, primers used to clone the upstream flanking region by PCR amplification are in pink and those used to clone the downstream flanking region are in purple. Primers used to sequence confirm the cloned flanking regions are in gray. Primers outside the flanking regions used to screen transformants for the deletion are in black. The tal3 and tal4 cluster deletions were unmarked. For the *tal5* gene deletion, the *cat* gene for chloramphenicol resistance (Cm^r) was used as a marker. It was cloned between the *tal5*-flanking regions into pK18mobSacB using overlap extension PCR. For this cloning, the 5' ends of the PCR primers for each fragment were designed complementary to the neighboring fragment sequence and are colored to match. White rectangles are sequences immediately flanking individual genes that were not used because they are not unique in the genome.

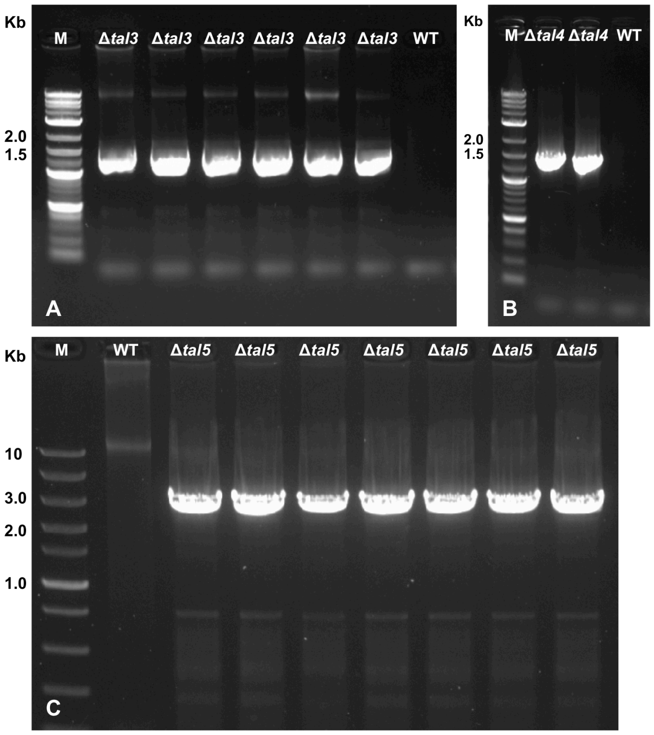

Figure S2. Molecular confirmation of the ICMP11055 tal3, tal4, and *tal5* deletion mutants identified. Genomic DNA of the mutants and the wild-type strain ICMP11055 was subjected to polymerase chain reaction (PCR) using primers outside the flanking regions used for the deletion mutagenesis in each case (see Supplementary Figure S1). M, 2log DNA Ladder (0.1-10.0 kb) in A and B, and Fast DNA Ladder (0.1-10.0 kb) in C (New England BioLabs); WT, wild type. The relatively large wildtype tal3 and tal4 clusters did not detectably amplify under the PCR conditions used.

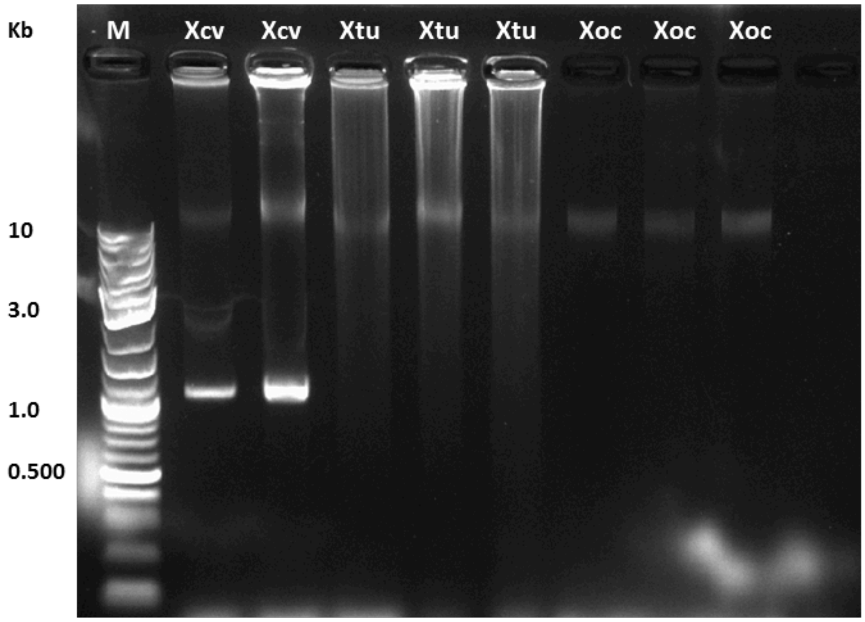

Figure S3. Assay for plasmids in ICMP11055 (Xtu) that would be missed by SMRT sequencing using a 20 kb library. *Xanthomonas campestris* pv. vesicatoria 85-10 (Xcv) (Thieme et al., 2005) and *Xanthomonas oryzae* pv. oryzicola BLS256 (Xoc) (Bogdanove et al., 2011) were included as a positive and negative control, respectively. Strains were cultured and DNA was extracted (independently twice for Xcv and three times each for Xtu and Xoc) and subjected to agarose gel electrophoresis as described by Chakrabarty *et al.,* (Chakrabarty et al., 2010). The leftmost lane contains 2-Log DNA Ladder (0.1-10.0 kb; New England BioLabs).

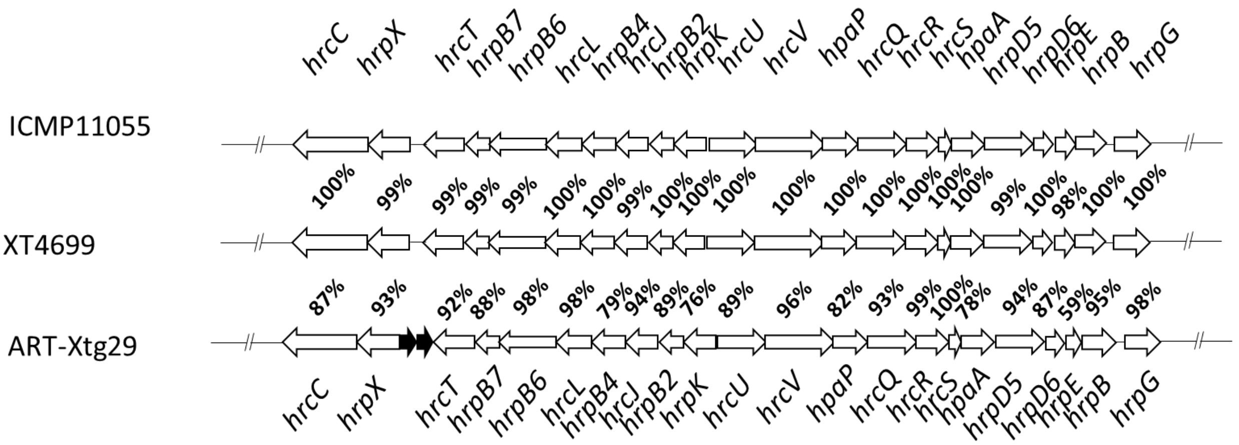

Figure S4. The type III secretion system (*hrp*/*hrc*) gene cluster of *X. translucens* pv. undulosa strain ICMP11055 relative to those of XT4699 and *X. translucens* pv. graminis strain ART-Xtg29. White arrows (drawn to scale) represent individual *hrp* or *hrc* genes, labeled at bottom. Above each XT4699 and ART-Xtg29 gene is shown the percent aa identity of the gene product to its counterpart in ICMP11055. Black arrows indicate two IS elements unique to the ART-Xtg29 cluster.

TALEs	1	2	3	4	5	6	7	8	9	10	11	12	13	14	15	16	17	18	
ICMP11055 Tal1	HD	YD	NI	NG	NG	NN	YK	NG	HD	NG	NG	ND	NG	QD	NH	HD			
XT4699 Tal1	HD	YD	NI	NG	NG	NN	YK	NG	HD	NG	NG	ND	NG	QD	NH	HD			
ICMP11055 Tal2	NN	HD	NG	NN	HN	KG	NI	HD	NI	HN	HD	HN	HD	Y*	NG	HD	HD	HN	
XT4699 Tal2	NN	HD	NG	NN	HN	KG	NI	HD	NI	NN	HD	HN	HD	HD	NI	HN	HD	QD	
XTRocky Tal2	NN	HD	NG	NN	HN	KG	NI	HD	NI	NN	HD	HN	HD	HD	NI	HN	--	HD	QD
DSM18974 TalCX1	NN	HD	NG	NI	HN	KG	NI	HD	NI	NH	NG	NN	HD	HD	NI	NN	NI	HD	QD
ICMP11055 Tal3b	HN	HD	HD	HD	NI	NI	NI	HN	HD	HD	NH	NN	NI	NN	HD				
XT4699 Tal7	HN	HD	HD	HD	NI	NI	NI	HN	HD	HD	NN	NN	NI	NN	HD				
XTRocky Tal7	HN	HD	HD	HD	NI	NI	NI	HN	HD	HD	NH	NN	NI	NN	HD				
P3 Tal7	HN	HD	HD	HD	NI	NI	NI	HN	HD	HD	NH	NN	NI	NN	HD				
CS4 Tal7	NN	HD	HD	HD	NI	NI	NI	HN	HD	HD	NH	NN	NI	NN	HD				
LG48 Tal7	HN	HD	HD	HD	NI	NI	NI	HN	HD	HD	NH	NN	NI	NN	HD				
LB5 Tla7	HN	HD	HD	HD	NI	NI	NI	HN	HD	HD	NI	NN	NI	NN	HD				
DSM18974 TalCT1	NN	HD	HD	HD	NI	NI	NI	NN	HD	HD	NN	NN	NI	NN	HD				
ICMP11055 Tal4a	HD	HN	HN	HD	NH	NH	HG	HD	KG	NN	Y*	NG	HD	HD	HN				
XT4699 Tal6	HD	HN	HN	HD	NH	NH	HG	HD	KG	NN	Y*	NG	HD	HD	HN				
XTRocky 3E3	HD	HN	HN	HD	NH	NH	H*	HD	KG	NN	Y*	NG	HD	NI	NH	NG	HD	HN	
ICMP11055 Tal4b	NN	HD	NG	HD	HD	HG	HD	KG	NN	Y*	NG	NG	HD	HD	QD	HN			
XT4699 Tal8	NN	--	NG	HD	HD	HD	--	KG	NN	Y*	NG	--	HD	HD	QD	HN			
XTRocky Tal8	NN	--	NG	HD	HD	HD	--	KG	NN	Y*	NG	--	HD	HD	QD	HN			
ICMP11055 Tal5	NH	NN	HD	NN	HD	NH	HD	YK	NG	NH	Y*	HD	NN	NI	NG	QD			
XT4699 Tal4	NH	NN	HD	NN	HD	NH	HD	YK	NG	NH	Y*	HD	NN	NI	NG	QD			
XT4699 Tal3	NN	HD	NG	HD	HD	HN	NF	NI	NH	HD	HD	HD	HN	HN	HD	--			
XT4699 Tal5	NN	HD	NG	NN	HN	HN	NI	NI	NH	NN	HD	NN	NH	NH	HD	HD			
ICMP11055 Tal3a	NN	HD	NG	HD	NG	HD	HD	HG	HD	KG	NN	KG	HD	HN	QD	HN	NN	HD	
CEBP2541 Tal1	NS	KI	NI	HD	NK	GI	HD	NK	HD	NN	HD	NK							
CEBP2541 Tal2	NN	NN	KI	NN	HD	NG	HD	NG	NG	NK	HD	HD	NN	QD	NG	QD			
DSM18974 TalCU1	NG	HD	HD	HN	NG	NI	HG	HG	HD	ND	NN	NN	NI	NH	QD				
DSM18974 TalCV1	NG	NN	HD	HD	NN	NI	HG	HD	ND	HG	NI	NN	HD						
DSM18974 TalCW1	NN	NI	HN	HD	NI	NH	NG	HN	HD	HD	HD	NI	QD						
DSM18974 TalCY1	NG	HN	NN	HD	NG	ND	ND	NK	QD	NH	QD								
DSM18974 TalCZ1	HD	NN	HD	NH	HD	YK	NG	NH	Y-	HD	NN								
DSM18974 TalDA1	NN	HD	NG	NG	NG	NN	YK	NG	HD	NG	NG	ND	NG	HD	NH	HD			

Figure S5. Alignment of repeat-variable di-residues (RVDs) of available *X. translucens* TALE sequences. RVDs that differ between otherwise conserved TALEs are shown in blue font. RVDs of unique TALEs are in red. An asterisk indicates that the second aa of the RVD is missing. Strains represented are Xtu strains ICMP11055, XT4699, XTRocky, P3, CS4, LG48, and LB5 (Peng et al., 2016), Xtc strain CFBP 2541 (Pesce et al., 2015), and Xtt strain DSM 18974 (Jaenicke et al., 2016).

A

B

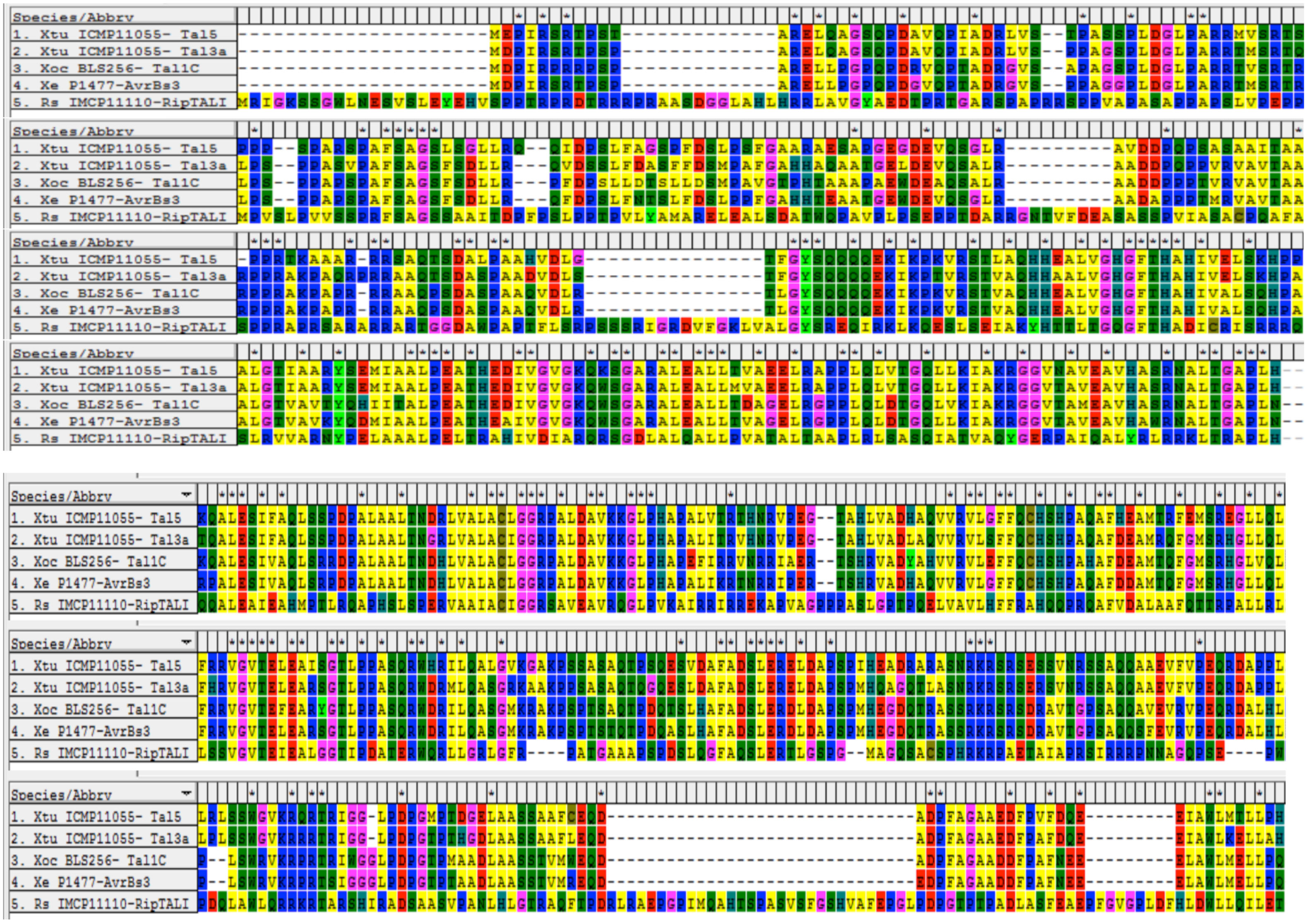

Figure S6. Alignment of the N-terminal (A) and C-terminal (B) amino acid sequences of Tal3a and Tal5 of ICMP11055, Tal1c of *Xanthomonas oryzae* pv. oryzicola BLS256, AvrBs3 of *Xanthomonas euvesicatoria* P1477, and RipTALI of *Ralstonia solanacearum* ICMP11110. Alignments were done using MEGA5.05.

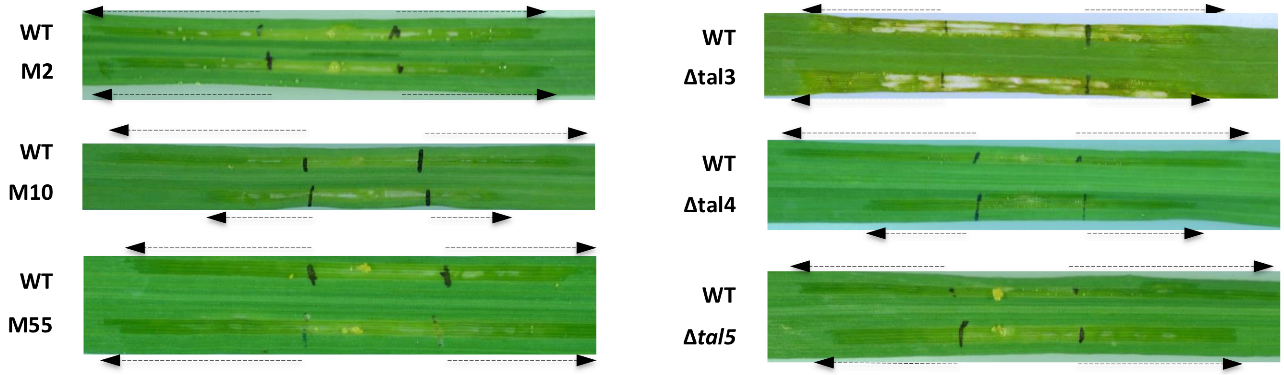

Figure S7. Representative disease symptoms on leaves of *Triticum aestivum* cv. Chinese Spring at 9 days post-inoculation with ICMP11055 wild type (WT) and *tal* mutant strains. Leaves were inoculated by infiltration using a syringe with a needle. Arrows mark the expansion of lesions past the infiltrated area.

References

Alizadeh, A., and Rahimian, H. (1989). Bacterial leaf streak of Gramineae in Iran. *EPPO Bull.* 19**,** 113-117. doi: 10.1111/j.1365-2338.1989.tb00136.x.

Bai, J., Choi, S.-H., Ponciano, G., Leung, H., and Leach, J.E. (2000). *Xanthomonas oryzae* pv. oryzae avirulence genes contribute differently and specifically to pathogen aggressiveness. *Mol. Plant-Microbe Interact.* 13**,** 1322-1329. doi: 10.1094/MPMI.2000.13.12.1322.

Bikard, D., Jiang, W., Samai, P., Hochschild, A., Zhang, F., and Marraffini, L.A. (2013). Programmable repression and activation of bacterial gene expression using an engineered CRISPR-Cas system. *Nucleic Acids Res.* 41**,** 7429-7437. doi: 10.1093/nar/gkt520.

Bogdanove, A.J., Koebnik, R., Lu, H., Furutani, A., Angiuoli, S.V., Patil, P.B., Van Sluys, M.-A., Ryan, R.P., Meyer, D.F., and Han, S.-W. (2011). Two new complete genome sequences offer insight into host and tissue specificity of plant pathogenic *Xanthomonas* spp. *J. Bacteriol.* 193**,** 5450-5464. doi: 10.1128/JB.05262-11.

Cermak, T., Doyle, E.L., Christian, M., Wang, L., Zhang, Y., Schmidt, C., Baller, J.A., Somia, N.V., Bogdanove, A.J., and Voytas, D.F. (2011). Efficient design and assembly of custom TALEN and other TAL effector-based constructs for DNA targeting. *Nucleic Acids Res.* 39**,** e82. doi: 10.1093/nar/gkr218.

Cernadas, R.A., Doyle, E.L., Niño-Liu, D.O., Wilkins, K.E., Bancroft, T., Wang, L., Schmidt, C.L., Caldo, R., Yang, B., and White, F.F. (2014). Code-assisted discovery of TAL effector targets in bacterial leaf streak of rice reveals contrast with bacterial blight and a novel susceptibility gene. *PLoS Path.* 10**,** e1003972. doi: 10.1371/journal.ppat.1003972.

Chakrabarty, P., Chavhan, R., Ghosh, A., and Gabriel, D. (2010). Rapid and efficient protocols for throughput extraction of high quality plasmid DNA from strains of *Xanthomonas axonopodis* pv. malvacearum and *Escherichia coli*. *J. Plant Biochem. Biotechnol.* 19**,** 99-102. doi: 10.1007/BF03323444.

Jaenicke, S., Bunk, B., Wibberg, D., Sproer, C., Hersemann, L., Blom, J., Winkler, A., Schatschneider, S., Albaum, S.P., Kolliker, R., Goesmann, A., Puhler, A., Overmann, J., and Vorholter, F.J. (2016). Complete genome sequence of the barley pathogen *Xanthomonas translucens* pv. translucens DSM 18974T (ATCC 19319T). *Genome Announc.* 4. doi: 10.1128/genomeA.01334-16.

Kvitko, B.H., and Collmer, A. (2011). "Construction of *Pseudomonas syringae* pv. *tomato* DC3000 mutant and polymutant strains," in *Plant Immunity*. Springer), 109-128.

Langlois, P.A., Snelling, J., Hamilton, J.P., Bragard, C., Koebnik, R., Verdier, V., Triplett, L.R., Blom, J., Tisserat, N.A., and Leach, J.E. (2017). Characterization of the *Xanthomonas translucens* complex using draft genomes, comparative genomics, phylogenetic analysis, and diagnostic LAMP assays. *Phytopathology* 107**,** 519-527. doi: 10.1094/PHYTO-08-16-0286-R.

Makino, S. (2005). *Molecular mechanisms of pathogenesis in pathovars of the rice pathogenic bacterial species Xanthomonas oryzae.* MS thesis Iowa State University.

Peng, Z., Hu, Y., Xie, J., Potnis, N., Akhunova, A., Jones, J., Liu, Z., White, F.F., and Liu, S. (2016). Long read and single molecule DNA sequencing simplifies genome assembly and TAL effector gene analysis of *Xanthomonas translucens*. *BMC Genomics* 17**,** 1. doi: 10.1186/s12864-015-2348-9.

Pesce, C., Bolot, S., Cunnac, S., Portier, P., Fischer-Le Saux, M., Jacques, M.-A., Gagnevin, L., Arlat, M., Noël, L.D., and Carrère, S. (2015). High-quality draft genome sequence of the *Xanthomonas translucens* pv. cerealis pathotype strain CFBP 2541. *Genome Announc.* 3**,** e01574-01514. doi: 10.1128/genomeA.01574-14.

Thieme, F., Koebnik, R., Bekel, T., Berger, C., Boch, J., Büttner, D., Caldana, C., Gaigalat, L., Goesmann, A., and Kay, S. (2005). Insights into genome plasticity and pathogenicity of the plant pathogenic bacterium *Xanthomonas campestris* pv. vesicatoria revealed by the complete genome sequence. *J. Bacteriol.* 187**,** 7254-7266. doi: 10.1128/JB.187.21.7254-7266.2005.

Verdier, V., Triplett, L.R., Hummel, A.W., Corral, R., Cernadas, R.A., Schmidt, C.L., Bogdanove, A.J., and Leach, J.E. (2012). Transcription activator-like (TAL) effectors targeting *OsSWEET* genes enhance virulence on diverse rice (*Oryza sativa*) varieties when expressed individually in a TAL effector-deficient strain of *Xanthomonas oryzae*. *New Phytol.* 196**,** 1197-1207. doi: 10.1111/j.1469-8137.2012.04367.x.

Wichmann, F., Vorhölter, F.J., Hersemann, L., Widmer, F., Blom, J., Niehaus, K., Reinhard, S., Conradin, C., and Kölliker, R. (2013). The noncanonical type III secretion system of *Xanthomonas translucens* pv. graminis is essential for forage grass infection. *Mol. Plant Pathol.* 14**,** 576-588. doi: 10.1111/mpp.12030.
